# Changes and driving factors of microbial community composition and functional groups during the decomposition of *Pinus massoniana* deadwood

**DOI:** 10.1002/ece3.11210

**Published:** 2024-04-01

**Authors:** Bingyang Shi, Xiurong Wang, Shuoyuan Yang, Hongmei Chen, Yang Zhao, Junjie Shen, Meixuan Xie, Bufang Huang

**Affiliations:** ^1^ Forestry College Guizhou University Guiyang Guizhou China

**Keywords:** bacterial community, community characteristics, decay classes, fungal community, *Pinus massoniana* deadwood

## Abstract

Clarifying changes in the microbial community in deadwood at different stages of decomposition is crucial for comprehending the role of deadwood in the biogeochemical processes and the sustainability of forest development. However, there have been no reports on the dynamics of microbial community during the decomposition of *Pinus massoniana*. We used the “space‐for‐time” substitution to analyze the characteristics of microbial community changes and the key influencing factors in the *P. massoniana* deadwood during different decomposition stages by 16S and ITS rRNA gene sequencing. The results suggest that the microbial community structure of the early decomposition (decay class I) was significantly different from the other decay classes, while the diversity and richness of the microbial community were the highest in the late decomposition (decay class V). The Linear Discriminant Analysis Effect Size analysis revealed that most bacterial and fungal taxa were significantly enriched in decay classes I and V deadwood. During the initial stages of decomposition, the relative abundance of the bacterial functional group responsible for carbohydrate metabolism was greater than the later stages. As decomposition progressed, the relative abundance of saprophytic fungi gradually decreased, and there was a shift in the comparative abundance of mixed saprophytic‐symbiotic fungi from low to high before eventually decreasing. Total organic carbon, total nitrogen, carbon‐to‐nitrogen ratio, total potassium, total phenol, condensed tannin, lignin, and cellulose were significantly correlated with microbial community structure, with the carbon‐to‐nitrogen ratio having the greatest effect. Our results indicate that the physicochemical properties of deadwood, microbial community structural composition and functional group changes were related to the decay class, among which the carbon‐to‐nitrogen ratio may be an important factor affecting the composition and diversity of microbial communities.

## Graphical abstract

Our results indicate that the physicochemical properties, microbial community structural composition and functional group changes were related to the decay class of deadwood, among which the carbon‐to‐nitrogen ratio may be an important factor affecting the composition and diversity of microbial communities

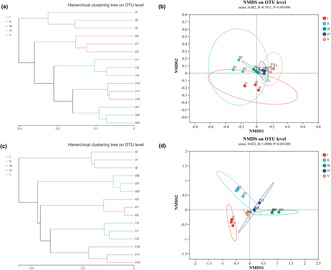

## 1 INTRODUCTION

Deadwood can provide crucial source of habitats and nutrients to a variety of organisms like bryophytes, microorganisms, and insects (Chang et al., 2014). With its abundant carbon reserves, energy, and nutrients, deadwood serves as an essential factor in the renewal and succession of forest ecosystems, the maintenance and conservation of biodiversity, as well as nutrient cycling and energy transfer (Magnússon et al., 2016; Přívětivý et al., 2016; Ricker et al., 2016). By undergoing decay and decomposition, deadwood releases nutrients like nitrogen and phosphorus, while also providing habitat and nutrient sources for various decomposers, thereby fulfilling important ecological roles in forest ecosystems (Baldrian et al., 2016; Ulyshen, 2016).

The microbial community plays a crucial role in regulating and influencing the rate and mechanism of deadwood decomposition (Sarah et al., 2016). For instance, fungi can promote the decomposition of wood by secreting enzymes that catalyze complex and recalcitrant macromolecules such as cellulose, hemicellulose, and lignin (Eichlerová et al., 2015; Peršoh, 2015). Bacteria also actively participate in the decomposition of wood (Tláskal et al., 2017) and are affected by relative humidity, pH, C/N ratio, and other factors during the decomposition process of deadwood (Baldrian, 2017; Hoppe et al., 2015). In the process of wood decomposition, bacteria and fungi may appear synergistic or antagonistic complex relationships, affecting wood decay rate and community function (Rinta‐Kanto et al., 2016). Extensive research has been conducted on the pivotal role of microorganisms in the decomposition of deadwood. For instance, Mieszkin et al. (2021) analyze the structure and function of the bacterial communities in the sapwood and the heartwood of oak trees, and find that the heartwood‐ and sapwood‐inhabiting bacterial communities significantly differed from one another in terms of richness and taxonomic composition. Similarly, Moll et al. (2018) analyze microbial communities in 13 different species of deadwood and observe significant differences in microbial OTU richness and community structure among various species as well as between sapwood and heartwood. Furthermore, Cui et al. (2019) explore the species diversity of wood‐decaying fungal communities on four types of conifers in the Greater and Lesser Khinggan Mountains, and find that different tree species have significant effects on the composition of the wood‐decaying fungal communities. The decomposition of deadwood is a dynamic process, with changes occurring in the microbial community characteristics as decomposition progresses. For example, Yuan et al. (2017) show that tree species were the main source of fungal community diversity at different decomposition stages, and fungal communities achieved the highest levels of diversity at the intermediate and late decomposition stages. Pioli et al. (2023) find that the α diversity of the bacterial community was primarily linked to the decomposition stages of the tree. Qi et al. (2023) observe the similarities in the microbial community structure and function between the early and late decomposition stages of deadwood, but differences in the middle stages. In summary, the type of wood, structural complexity (sapwood and heartwood), and decomposition stage of deadwood are closely associated with shifts in microbial communities. However, the relationship between microbial communities and deadwood decomposition remains unclear due to various influencing factors (tree species, wood structure, decomposition stage, etc.), necessitating further extensive studies for supplementation and validation.

*Pinus massoniana* Lamb. is the most widely distributed native industrial timber species in the genus *Pinus* in China, and a crucial dominant species in Guizhou province (Luo & Yu, 2021). The average annual afforestation area of Guizhou province amounts to 200,000 hm^2^, with *P. massoniana* forests accounting for approximately 1/4–1/3 of the overall area (Li et al., 2016). The formation of deadwood of *P. massoniana* is attributed to the succession and renewal of forest ecosystems, and anthropogenic and natural disturbances. In addition, with the process of decomposition, the deadwood resources will eventually be transformed into soil (Mäkipää et al., 2017), which will further affect the soil nutrient cycle and the forest carbon sink function under the *P. massoniana* forest. In recent years, despite the large number of scholars who have conducted research on *P. massoniana* in Guizhou province, the majority have focused only on the relationship between *P. massoniana* forest stand, stand age, community plant diversity, and growing environment (Guo et al., 2019; Li et al., 2016; Luo & Yu, 2021; Ni et al., 2017) and have neglected research on *P. massoniana* deadwood. In addition, to the best of our knowledge, there have been no reports on the dynamics of microbial community during the decomposition of *P. massoniana*. Given that *P. massoniana* is a conifer tree, we may presume that its deadwood decomposition process shares similarities with that of other coniferous species. Nevertheless, these assumptions require further empirical validation. Therefore, we assert that examining the decomposition patterns and microbial community dynamics of *P. massoniana* during the decay process is a necessary undertaking.

As the decomposition of deadwood is a protracted process, it cannot be comprehensively studied within a short duration. “Space‐for‐time” substitution is widely used in biodiversity modeling to infer past or future trajectories of ecological systems from contemporary spatial patterns (Blois et al., 2013). Based on this, employed different decay classes of deadwood as a proxy to forecast changes anticipated during the decomposition process. To analyze bacterial and fungal community composition in *P. massoniana* deadwood, we employed the Illumina MiSeq high‐throughput sequencing technology. The main objectives were to (1) clarify the changing patterns of bacterial and fungal community composition and diversity of *P. massoniana* deadwood at different decomposition stages; (2) investigate structural modifications in major functional groups and microbial communities during each stage; and (3) delineate the critical factors that influence shifts in microbial community structure and diversity. The results provide guidance and reference for revealing the decomposition law of *P. massoniana* deadwood and the dynamic change characteristics of the microbial community in the decomposition process. Our work is crucial in comprehending biogeochemical processes and forest sustainability. Moreover, further research on the structure and function of microbial communities at different decomposition stages will help to discern their functional importance to deadwood decay.

## 2 MATERIALS AND METHODS

### 2.1 Study site and material sampling

Our research area was located in Guiyang, Guizhou Province, characterized by a subtropical humid and mild climate with plateau monsoon climate features. The local average annual temperature was 15.3°C, the average annual relative humidity was 77%, the average annual total precipitation was 1129.5 mm, and the average annual sunshine hours was 1148.3 hours. In November 2021, samples of *P. massoniana* deadwood (Figure S1) were collected from the long‐term protection and monitoring site of natural *P. massoniana* forest in Huaxi District, Guiyang City (106°39′10.21″–106°39′14.89″E, 26°26′43.44″–26°26′59.46″N), with an altitude of 1138.31–1164.09 m, covering an area of about 15.33 ha. The soil was subtropical yellow soil (Guo et al., 2019), and the vegetation was mainly coniferous forest with *P. massoniana* as the dominant species.

Deadwood was classified into five decay classes (I, II, III, IV, and V) following the criteria of Yan et al. (2005) (Table S1). We chose deadwood within the 20–40 cm diameter range, with similar site conditions and in contact with the ground as the research object. Three deadwoods were selected for each decay class, and a total of 15 deadwoods were collected (3 replicate deadwoods × 5 decay classes). Given the difficulty in accurately differentiating the bark, sapwood, and heartwood of decay classes IV and V, a composite sampling approach was adopted for all decay classes from I to V. We radially cut the deadwood into 4 cm pieces, removed surface impurities, placed them in sterile bags, and immediately stored them in a 4°C incubator for low‐temperature storage. Then they were cryogenically ground by mortar and passed through a 10 mm sieve and mixed well. A portion was used for the determination of the physical and chemical properties of the matrix after air‐drying, and the other portion was ground and passed through a 2 mm sieve and then stored at −80°C for the determination of microbial diversity.

### 2.2 DNA extraction, PCR amplification, and Illumina sequencing

The microbial DNA was extracted from deadwood samples utilizing the FastDNA kit (MP Biomedicals, USA). The bacterial 16S rRNA gene targeting the V3‐V4 region was amplified with 338F (ACTCCTACGGGAGGCAGCAG) and 806R (GGACTACHVGGGTWTCTAAT) (Liu et al., 2016). The fungal Internal Transcribed Spacer gene was amplified with primer pairs: ITS1F (CTTGGTCATTTAGAGGAAGTAA) and ITS2R (GCTGCGTTCTTCATCGATGC). The PCR reaction mixture (20 μL) contained 4 μL of 5 × FastPfu Buffer, 2 μL of 2.5 mM dNTPs, 0.8 μL of 5 μM Primer F, 0.8 μL of 5 μM Primer R, 0.4 μL of DNA Polymerase, 0.2 μL of BSA, 10 ng of Template DNA. PCR was carried out on a GeneAmp PCR System 9700 (Applied Biosystems, Foster City, CA, USA) utilizing the following program: an initial denaturation of 3 min at 95°C, 30 cycles of denaturation for 30 s at 95°C, annealing for 30 s at 55°C, and extension for 45 s at 72°C, and a single extension at 72°C for 10 min, 10°C until halted by the user. Subsequently, PCR products were extracted from 2% agarose gels and purified using an AxyPrepDNA Gel Extraction Kit (Axygen Biosciences, Union City, CA, USA). The purified amplification products were mixed in equal molarity, and Miseq libraries were constructed and sequenced using the Illumina MiSeq PE300 platform. The raw reads were uploaded to the NCBI Sequence Read Archive database (Accession Number: PRJNA905865).

### 2.3 Processing and analyzing of sequencing data

Fastq (https://github.com/OpenGene/fastp, version 0.20.0) was used to demultiplex and quality filter raw sequence files (Chen et al., 2018), and FLASH (http://www.cbcb.umd.edu/software/flash, version 1.2.7) was used to merge them (Magoc & Salzberg, 2011). A 50 bp window was set up to filter bases with quality scores below 20 in the tail of reads. The truncated reads were shorter than 50 bp and the reads containing ambiguous characters were removed. According to the overlap between PE reads, pairs of reads were merged into a sequence with minimum overlap length of 10 bp. The overlap region's maximum mismatch ratio was 0.2. The samples were distinguished based on the barcode and primers, the sequence orientation was modified, the number of mismatches allowed by the barcode was set to 0, and the maximum number of primer mismatches was set to 2. Using UPARSE software (version 7.1), according to the similarity of 97% to OTU sequence clustering, and chimeric sequences were discovered and discarded (Edgar, 2013). Taxonomic annotation of OTU species was performed by comparing the Silva 16S rRNA gene database (v138) and ITS database (Unite 8.0) with the RDP classifier (version 2.11), with a confidence threshold of 70% (Wang et al., 2007). Finally, OTU species classification tables were generated by removing unclassified species at the phylum level and those with a total number of sequences <20.

### 2.4 Determination of physical and chemical properties of deadwood

The pH of 1:1 wood‐water suspension was measured using a FieldScout soil in situ pH meter—PH400 and PH600 (Spectrum, USA). Total nitrogen (TN) content was determined by the semi‐trace Kjeldahl method, total phosphorus (TP) content was measured by molybdenum‐antimony anti‐colorimetry method, and total potassium (TK) content was measured by flame spectrophotometry method, total organic carbon (TC) content was determined by the concentrated sulfuric acid‐potassium dichromate method (Liu, Yang, et al., 2022; Shi et al., 2022). Total phenol (*T*_p_) was measured by the colorimetric method of Folin‐Ciocalteau, and condensed tannin (*C*_t_) was measured by the vanillin‐hydrochloric acid method (Fan et al., 2018). The lignin (*X*_y_) content was determined using the Solarbio Lignin Content Assay Kit (trace method) (Zhu et al., 2023). The cellulose (*C*_e_) content was determined using the Solarbio Lignin (BC4280) Content Assay Kit (Spectrophotometer method).

### 2.5 Statistical analysis

Bioinformatics analysis was performed using the microbial diversity cloud from the online platform (https://cloud.majorbio.com/) of Shanghai Meiji Biomedical Technology (Ren et al., 2022). Based on the OTU table obtained above, rarefaction curves were generated using “alpha_rarefaction.py” in QIIME to calculate the alpha diversity indices (Zhou et al., 2022). Alpha diversity analysis involved the evaluation of microbial community richness using the Chao1 and Ace indices, with higher values indicating increased community richness. Microbial community diversity was estimated through the Shannon diversity index and Simpson index, with a larger Shannon index indicating higher community diversity, while a lower Simpson index indicated greater diversity. For beta diversity, hierarchical cluster dendrograms were generated using Mothur based on the OTU composition to compare the bacterial and fungal communities of all deadwood samples (based on Bray–Curtis) (R software: version 3.3.1) (Gu et al., 2021). The degree of dissimilarity among bacterial or fungal communities for all samples was determined using nonmetric multidimensional scaling (NMDS) at the OTU level (based on weighted UniFrac) (R software: version 3.3.1), and ANOSIM was used to detect intra‐group and inter‐group differences (999 permutations) (Shen et al., 2022).

Linear discriminant analysis (LDA) coupled with effect size measurements (LEfSe) analysis was conducted to search for significantly different taxa of microorganisms between the deadwood samples. The significance criteria for both bacteria and fungi at the taxonomic level were LDA > 3.5 and *p* < .05 (Liu, Li, et al., 2022). The relationship between environmental variables and microbial communities in deadwood samples was studied by Canonical Correspondence Analysis (CCA) and Redundancy Analysis (RDA). The selection of RDA or CCA models was based on the results of Detrended Correspondence Analysis (DCA) using the species‐sample data (sample OTU table with 97% similarity), where the size of the first axis of the gradient was considered. If it was ≥3.5, CCA was selected, but if it was <3.5, the result of RDA was better than CCA. In addition, Spearman correlation heat maps were employed to estimate the relationship between bacterial and fungal communities at the phylum level and environmental variables. At the OTU level, the bacterial ecological functional groups were analyzed with the PICRUSt2 package (2.2.0) (https://github.com/picrust/picrust2) in combination with the KEGG database and the EggNOG databases (Douglas et al., 2020; Wang et al., 2022). Fungal ecological functional groups were analyzed with FUNGuild, whereby the confidence level of the functional prediction results was categorized as highly probable, probable, and possible (Jiang et al., 2021). The communities that could not be identified or identified as multiple complex nutrition methods were unified as “undefined”. The experimental data were statistically analyzed with Excel 2010 and IBM SPSS Statistics 19 software, and one‐way analysis of variance (ANOVA) and Tukey's HSD were used to analyze the differences and significance of the physical and chemical properties of the deadwood.

## 3 RESULTS

### 3.1 Microbial diversity and community composition of *P. massoniana* deadwood at different stages of decomposition

**FIGURE 1:**
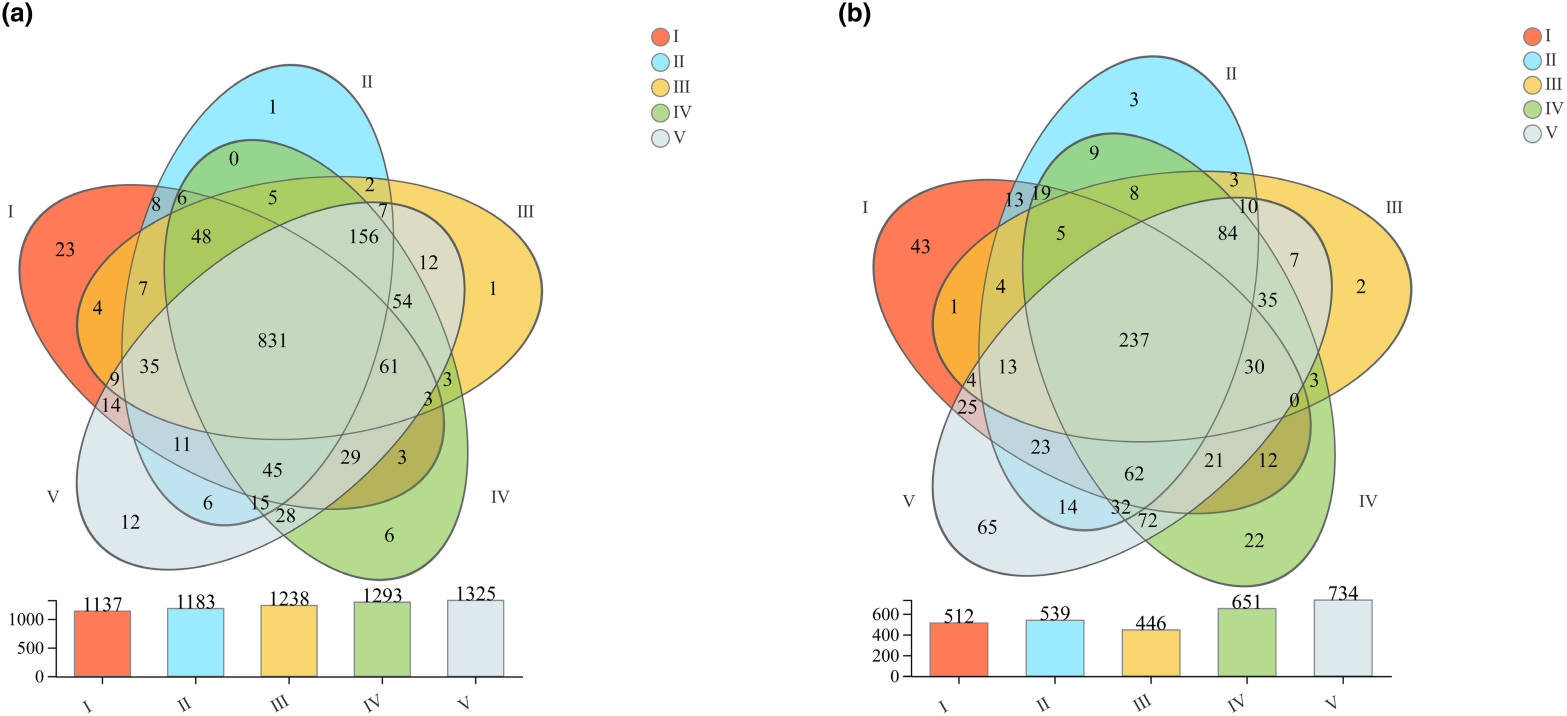
Venn diagram of bacterial (a) and fungal (b) communities describing the number of shared OTUs among the deadwood with different decay classes.

16S rRNA gene and ITS rDNA sequencing of microbial communities in deadwood at different decomposition stages were carried out. After optimizing and filtering out the low‐quality sequences, we obtained 813,985 effective sequences, 336,966,737 effective sequence bases, and 413 bp average length of bacteria 16S rRNA fragments in the deadwood samples. The effective sequences and effective sequence bases number of the fungal ITS rDNA fragments were respectively 1,057,308 and 260,743,295 with an average length of 246 bp. These sequences were resolved and removed from redundancy, and then subjected to OTU clustering at 97% similarity. Finally, the unclassified species at the phylum level and the species with low sequence numbers were removed to obtain 1445 bacterial and 881 fungal OTUs, respectively. The total number of bacterial OTUs in deadwood with different decay classes showed that decay class V (1325) > decay class IV (1293) > decay class III (1238) > decay class II (1183) > decay class I (1137), where the number of identical OTUs in each decay class was 831 and the number of unique OTUs was 23 for decay class I, 1 for decay class II, 1 for decay class III, 6 for decay class IV, and 12 for decay class V (Figure 1a). The total number of fungal OTUs was: decay class V (734) > decay class IV (651) > decay class II (539) > decay class I (512) > decay class III (446), where the number of identical owned OTUs was 237 and the unique OTUs was 43 for decay class I, 3 for decay class II, 2 for decay class III, 22 for decay class IV, and 65 for decay class V. (Figure 1b). The rarefaction curves based on OTU tended to be flat and saturated, which indicated that the amount sampling and testing data was reasonable. The results can truly and accurately reflect the bacterial and fungal communities in deadwood samples within each different decay class (Figure S1).

Alpha diversity analysis showed that the bacterial diversity of decay class V was the highest, and that of decay class II was the lowest, and there was a significant difference between them (*p* < .05). There was no significant difference in bacterial diversity between decay classes II, I, and III. In the fungal community, the fungal diversity of decay class V was the highest, which was significantly higher than that of decay classes II and III (*p* < .05), but there was no significant difference between decay classes V and IV. According to the analysis of the Chao1 index and Ace index, the bacterial richness of decay class V was the highest and significantly higher than that of decay classes I and II (*p* < .05), but had no significant difference with decay classes III and IV. The fungal richness of decay class V was the highest, and significantly higher than that of other decay classes (*p* < .05). In conclusion, compared with other decay classes, decay class V showed the highest microbial community diversity and richness. With the decomposition of deadwood, the diversity and richness of the microbial community increased gradually (Table 1).

**TABLE 1:** Alpha diversity indices of bacterial and fungal communities of deadwood with different decay classes.

Deadwood	Bacterial community				Fungal community			
	Shannon	Simpson	Ace	Chao1	Shannon	Simpson	Ace	Chao1
I	5.30 ± 0.37 ab	0.018 ± 0.0164 a	1046.92 ± 69.98 c	1048.63 ± 69.68 c	3.35 ± 0.29 b	0.097 ± 0.0151 bc	447.69 ± 28.09 c	451.36 ± 29.24 c
II	4.81 ± 0.45 b	0.034 ± 0.0201 a	1130.94 ± 26.09 bc	1128.85 ± 8.02 bc	2.35 ± 0.36 c	0.329 ± 0.1019 a	490.10 ± 18.43 c	470.75 ± 22.74 c
III	5.48 ± 0.19 ab	0.012 ± 0.0062 a	1205.16 ± 43.92 ab	1201.80 ± 64.73 ab	2.14 ± 0.08 c	0.208 ± 0.0095 ab	468.98 ± 49.83 c	413.65 ± 19.13 c
IV	5.57 ± 0.06 a	0.010 ± 0.0005 a	1239.63 ± 12.61 ab	1245.84 ± 9.08 ab	3.90 ± 0.46 ab	0.054 ± 0.0312 c	574.25 ± 32.44 b	575.51 ± 32.38 b
V	5.75 ± 0.08 a	0.010 ± 0.0010 a	1267.42 ± 26.70 a	1283.36 ± 27.39 a	4.42 ± 0.22 a	0.038 ± 0.0112 c	665.58 ± 14.32 a	675.05 ± 18.89 a

*Note*: Data were presented as the mean ± standard error (*n* = 3). Different lowercase letters in the same row indicated significant difference (*p* < .05) (one‐way ANOVA and Tukey's HSD test).

**FIGURE 2:**
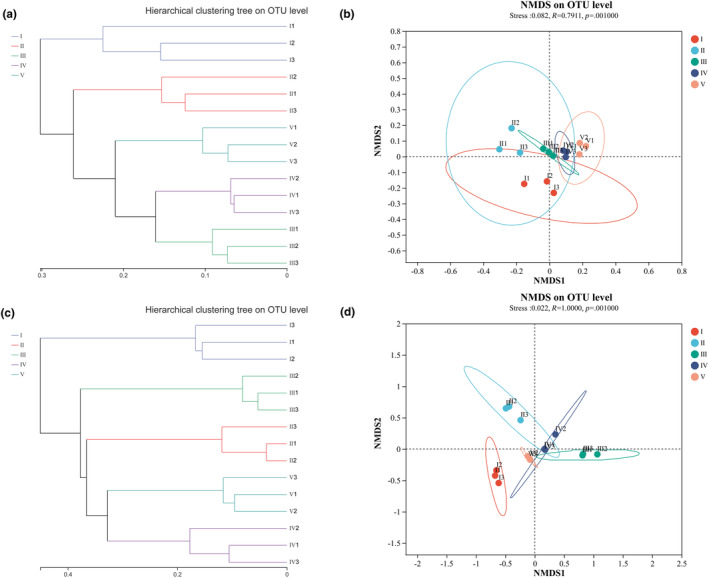
Beta diversity of bacterial (a, b) and fungal communities (c, d) of deadwood with different decay classes. Hierarchical clustering of bacterial and fungal communities based on Bray‐Curtis distance metrics (a, c) and NMDS of bacterial and fungal communities based on weighted UniFrac distance metrics (b, d).

Based on the Bray‐Curtis distance hierarchical clustering analysis, the five decay classes of deadwood samples in the bacterial and fungal communities were mainly divided into two major clusters (Figure 2a,c). Cluster 1 consisted of the three samples of decay class I deadwood, which was significantly different from other decay classes of deadwood. In the bacterial community, cluster 2 could be grouped into two subclusters, among which the decay class II deadwood cluster was one subcluster, and decay classes III, IV, and V deadwood belonged to another subcluster (Figure 2a). In the fungal community, cluster 2 was also grouped into two subclusters, among which decay class III deadwood was grouped into one subcluster, and decay classes II, IV, and V deadwood were grouped into another subcluster (Figure 2c). The results of weighted UniFrac NMDS analysis on OTU abundance showed that decay class I deadwood samples were significantly separated from other decay classes of deadwood samples in the bacterial and fungal communities (Figure 2b,d). The results were similar to those of hierarchical clustering, indicating that there were significant differences in the microbial community structure between decay class I deadwood (at the early decomposition stage) and other decay classes.

**FIGURE 3:**
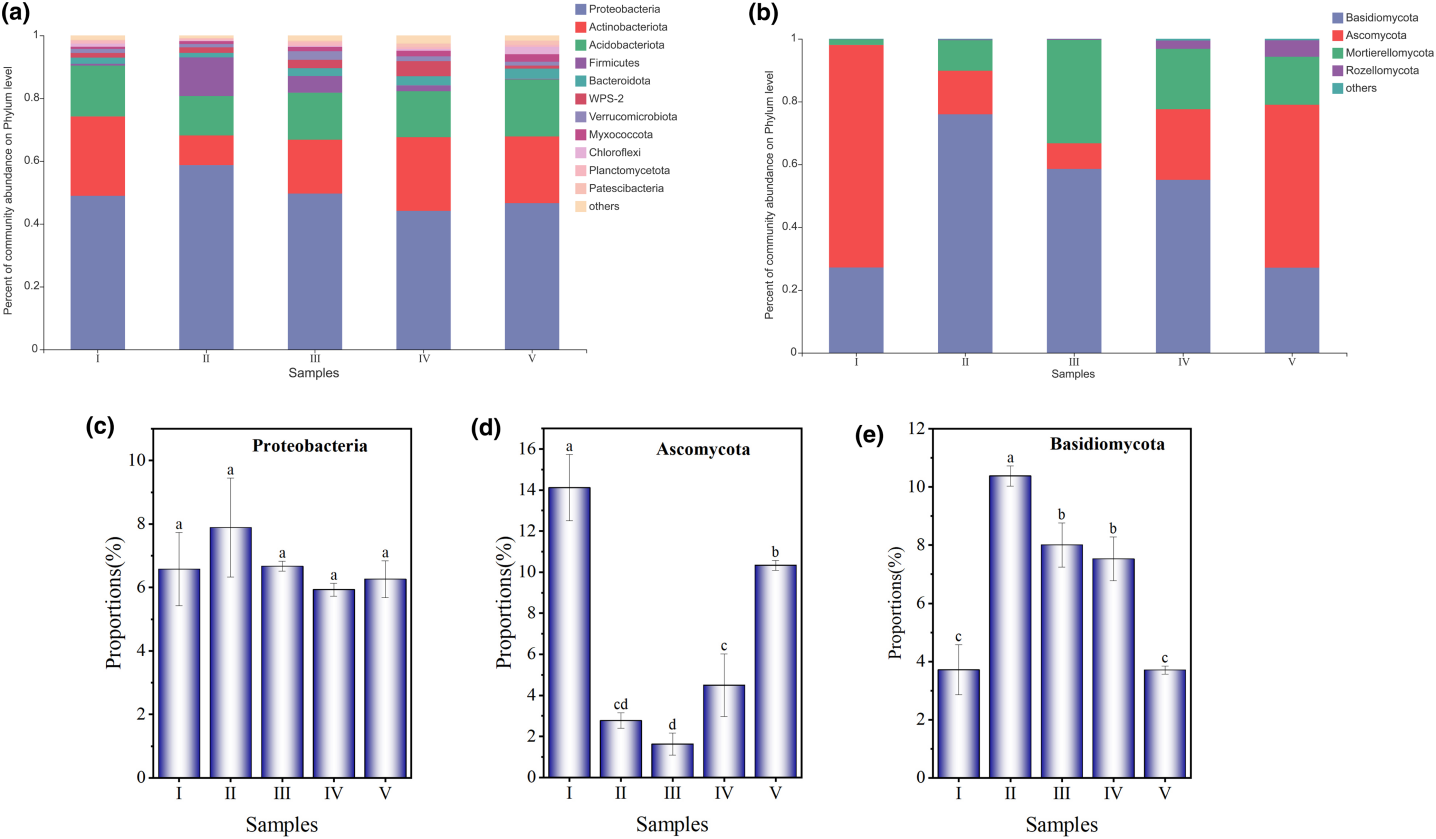
Relative abundances of different bacterial taxa (a) and fungal taxa (b) at the phylum level and major variations. (c–e) represent the comparison between the relative abundance of dominant microorganisms in different decay classes (one‐way ANOVA and Tukey's HSD test). Phyla representing less than 1% of the total reads are grouped as “Others”.

Based on the OTU taxonomic affiliation at a 97% similarity level, the bacterial communities in different decay classes of deadwood contained 25 phyla, 58 classes, 142 orders, 232 families, 392 genera, and 688 species. The most abundant bacterial phyla were *Proteobacteria* (44.14–58.70%), *Actinobacteriota* (9.45–25.26%), *Acidobacteriota* (12.56–18.16%) and *Firmicutes* (0.20–12.30%), *Bacteroidota* (1.47–3.24%), and *WPS‐2* (0.95–4.83%), accounting for more than 90% of the sequences in all groups (Figure 3a). Among them, *Proteobacteria* (44.1–58.7%) was the dominant bacteria phylum, but its relative abundance has no significant difference among all decay classes (Figure 3c). The fungal community contained 398 species, 276 genera, 160 families, 82 orders, 10 phyla, and 34 classes. The fungal phylum was dominated by *Basidiomycota* (27.17–75.96%), *Ascomycota* (8.16–70.86%), *Mortierellomycota* (1.79–32.93%), and *Rozellomycota* (0.03–5.23%). Among them, *Ascomycota* (8.16–70.86%) dominated in decay classes I and V deadwood, and its relative abundance in decay class I was significantly higher than that in other decay classes. *Basidiomycota* (27.17–75.96%) dominated in decay classes II, III, and IV deadwood (Figure 3b), and its relative abundance in decay class II was significantly higher than that in other decay classes.

**FIGURE 4:**
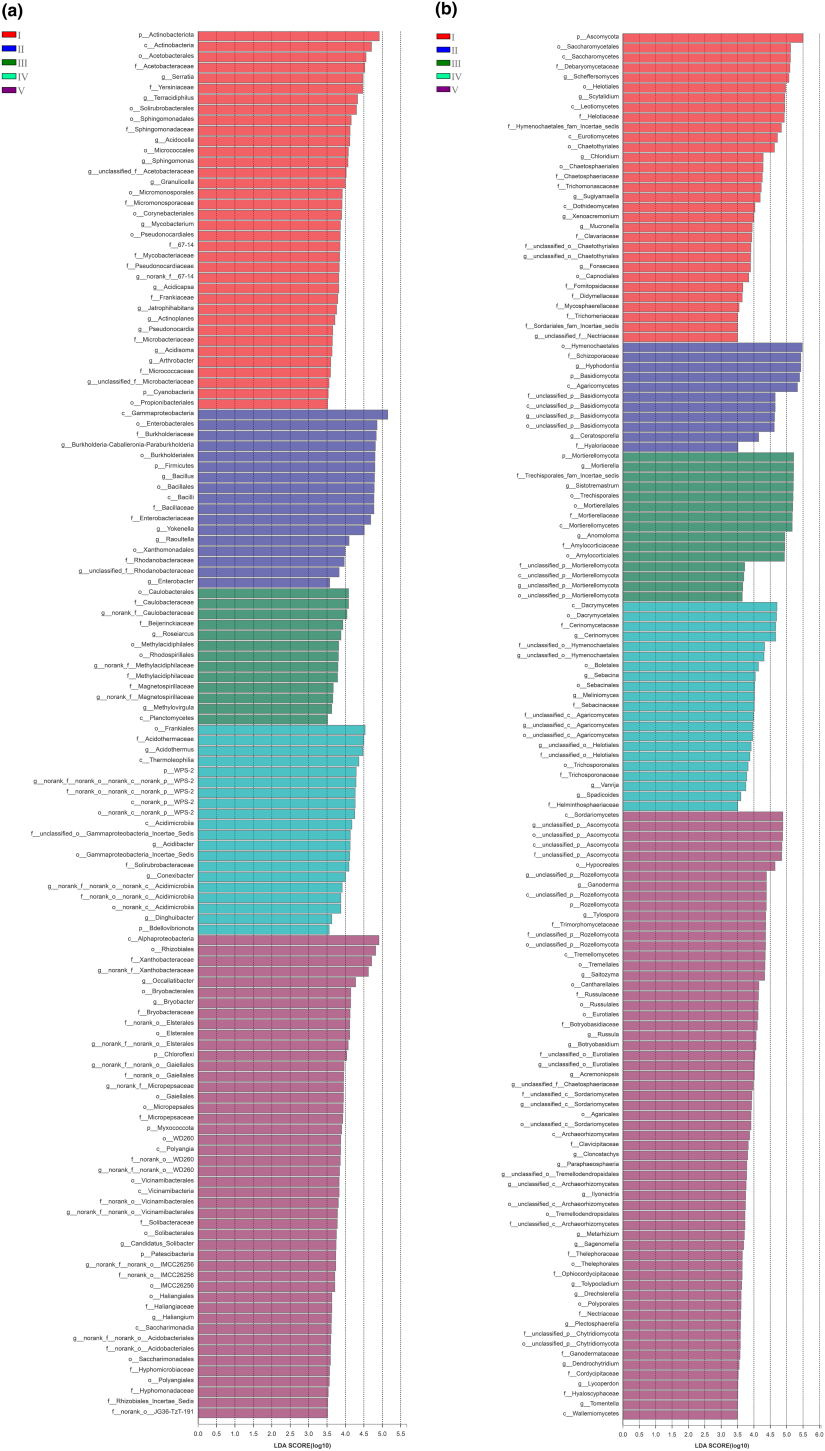
Bacterial (a) and fungal (b) diversity of deadwood with different decay classes were analyzed by LefSe; the levels of phylum, class, order, family, and genus were indicated with p, c, o, f, and g, respectively, before the name of each microorganism. Only taxa with a *p* < .05 and an LDA score over 3.5 is shown.

We performed LEfSe analysis for LDA scores above 3.5 (Figure S3), and found that most bacterial and fungal taxa were significantly enriched in decay classes I and V deadwood (*p* < .05) (Figure 4, Figure S3). There were 17 bacterial taxa and 14 fungal taxa significantly enriched in decay class I deadwood, and they were mainly distributed in the dominant phyla *Acidobacteriota* (11 species), *Proteobacteria* (5 species), *Ascomycota* (11 species), and *Basidiomycota* (3 species), with one species of bacteria distributed in the non‐dominant phylum *Cyanobacteria* (Figure 4). There were 19 bacterial taxa and 28 fungal taxa significantly enriched in decay class V deadwood, and they were mainly distributed in the dominant phyla *Actinobacteriota* (7 species), *Proteobacteria* (8 species), *Ascomycota* (16 species), *Basidiomycota* (9 species), and *Rozellomycota* (1 species), with four species of bacteria distributed in the non‐dominant phylum *Myxococcota* (2 species), *Chloroflexi* (1 species), and *Patescibacteria* (1 species), and two species of fungal distributed in the non‐dominant phylum *Chytridiomycota* (Figure 4). In addition, 6 bacterial taxa and 4 fungal taxa were significantly enriched in decay class II deadwood, 7 bacterial taxa and 4 fungal taxa were significantly enriched in decay class III deadwood, and 8 bacterial taxa and 9 fungal taxa were significantly enriched in decay class IV deadwood (Figure 4).

### 3.2 Functional prediction of the microbial community of deadwood

**FIGURE 5:**
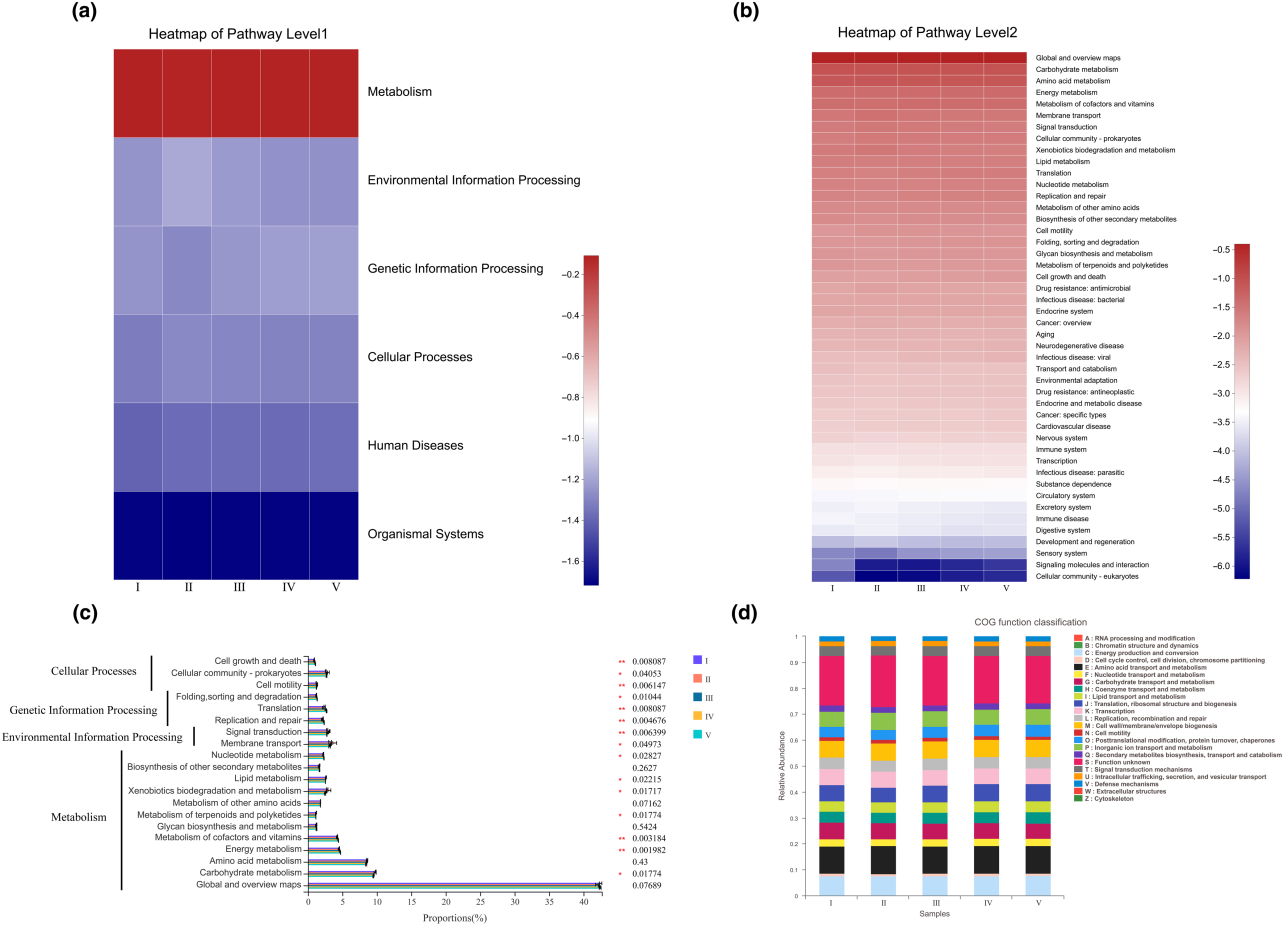
Functional predictions of deadwood with different decay classes microbiotas. (a, b) represented functional genes annotated in the KEGG database at levels 1–2, respectively; (c) represented variations in the relative abundance of KEGG pathway (level 2) genes (the relative abundance was >1%); (d) represented clusters of Orthologous Groups of proteins (COG) functional categories.

The differences in microbial community composition and diversity in different decomposition stages of deadwood might lead to changes in microbial community function. In order to determine the differences in microbial community functions, the PICRUSt2 analysis tool was used to predict the relative abundance of different metabolic pathways based on 16S rRNA sequencing data. Among them, the first‐level functional layer obtained six types of biological metabolic pathway functions (cellular processes, environmental information processing, human diseases, metabolism, organismal systems, and genetic information processing), while metabolism (76.97–78.13%) was the primary function (Figure 5a). Analysis of its second functional layer found that it consisted of 46 subfunctions. Among them, the relative abundance of 20 secondary functional layers was >1% (Figure 5b). Global and overview maps were dominant, followed by carbohydrate metabolism and amino acid metabolism, and the relative abundances of other functions were all lower than 5%. In the early stages of decomposition, the relative abundance of the bacterial functional group involved in carbohydrate metabolism was greater than in the later stages. Among the 20 secondary functional layers, the relative abundance of the 15 metabolic pathways was significantly different (*p* < .05) except for the other 5: glycan biosynthesis and metabolism, biosynthesis of other secondary metabolites, metabolism of other amino acids, amino acids metabolism, and global and overview maps (Figure 5c). COG (Orthologous Groups of proteins) functional analysis revealed a total of 23 protein homology groups in five decay classes, of which, 18.41 to 19.84% of COG functions were unknown, accounting for the highest proportion, followed by amino acid transport and metabolism (10.45–10.75%), energy production and conversion (7.53–7.68%), and cell wall/membrane/envelope biogenesis (6.44–6.67%) (Figure 5d).

**FIGURE 6:**
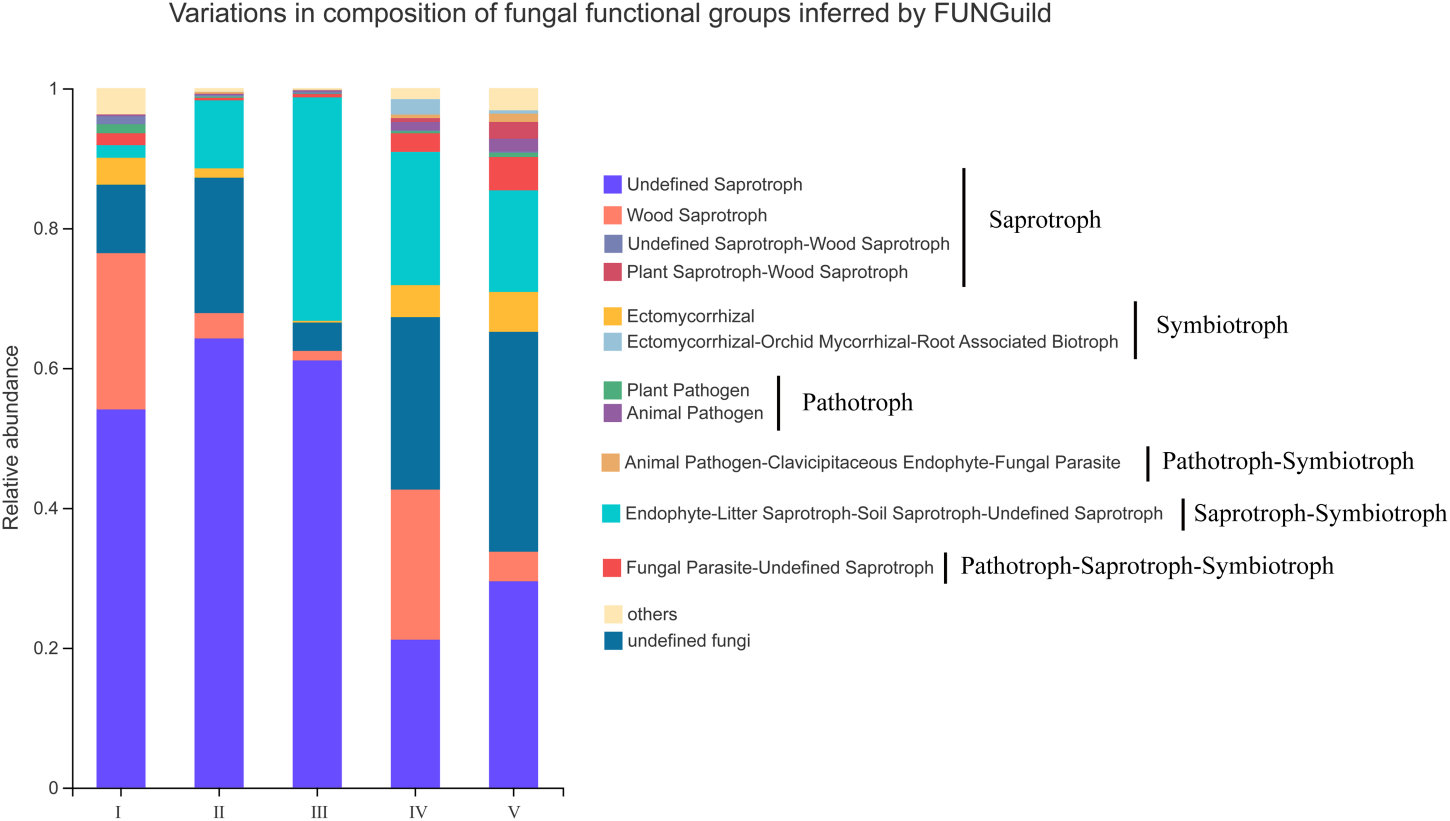
Normalized relative abundance of deadwood with different decay classes fungal functional.

Based on the FunGuild database, the trophic types of the fungi community were identified, and it was found that saprotroph and saprotroph‐symbiotroph were the main types (Figure 6). Saprophytic type was the absolute dominant type, and *Ascomycota* was the absolute dominant phylum. However, with the increase in decay class, the relative abundance of saprotrophic fungi gradually decreased, and the relative abundance changes of saprophyte‐symbiotic fungi showed low – high – low. *Mortierellomycota* was the dominant phylum of the saprophyte‐symbiotic type. According to the absorption and utilization of environmental resources, undefined saprotroph was the main species in each decay class of deadwood, and saprotroph‐symbiotroph was more relatively abundance in the decay classes III, IV, and V compared with the decay classes I and II.

### 3.3 Driving factors affecting microbial community change

In order to explore how environmental factors affect the microbial community in deadwood, we measured and analyzed the physical and chemical properties of deadwood. It was found that the total carbon (TC), C/N ratio (TC/TN), and cellulose content (*C*_e_) of the deadwood decreased gradually with the progress of decomposition (from decay classes I to V), while the contents of total nitrogen (TN), total potassium (TK), total phenol (*T*_p_), and lignin (*X*_y_) increased gradually (Table 2). In addition, there was no significant difference in total phosphorus (TP) content among the five decay classes of deadwood. The pH of decay classes I and II were significantly higher than that of decay classes III and IV (*p* < .05), while the pH of decay class V was the highest. The content of condensed tannin (*C*_t_) in decay classes I, IV, and V was significantly higher than that of decay classes II and III (*p* < .05).

**TABLE 2:** Comparison of physical and chemical properties of deadwood with different decay classes.

Deadwood	TC (g/kg)	TN (g/kg)	TC/TN	TP (g/kg)	TK (g/kg)	pH	*T*_p_ (g/kg)	*C*_t_ (g/kg)	*C*_e_ (g/kg)	*X*_y_ (g/kg)
I	45.95 ± 2.44 a	0.25 ± 0.03 c	187.68 ± 22.27 a	1.68 ± 0.11 a	0.28 ± 0.01 d	4.86 ± 0.01 b	15.05 ± 0.87 d	1.46 ± 0.22 a	43.73 ± 1.38 a	421.52 ± 16.18 b
II	44.42 ± 2.05 ab	0.39 ± 0.03 b	115.79 ± 11.75 b	1.31 ± 0.26 a	0.59 ± 0.03 c	4.91 ± 0.07 b	18.00 ± 0.63 c	0.46 ± 0.06 b	33.10 ± 6.10 b	425.50 ± 17.96 b
III	41.04 ± 1.11 bc	0.42 ± 0.05 b	99.21 ± 8.64 bc	1.70 ± 0.22 a	0.85 ± 0.02 b	4.56 ± 0.01 c	17.91 ± 0.97 c	0.59 ± 0.14 b	20.84 ± 5.08 c	506.61 ± 47.68 a
IV	39.79 ± 1.06 bc	0.49 ± 0.05 ab	81.03 ± 6.43 cd	1.64 ± 0.20 a	0.84 ± 0.03 b	4.50 ± 0.03 c	21.56 ± 1.27 b	1.31 ± 0.22 a	12.78 ± 3.04 cd	555.93 ± 2.99 a
V	36.56 ± 1.58 c	0.62 ± 0.07 a	59.93 ± 8.64 d	1.78 ± 0.15 a	1.14 ± 0.05 a	5.03 ± 0.02 a	26.90 ± 0.94 a	1.25 ± 0.31 a	3.25 ± 0.92 d	562.19 ± 30.54 a

*Note*: Data were presented as the mean ± SE (*n* = 3). Different lowercase letters in the same row indicated significant difference (*p* < .05) (one‐way ANOVA and Tukey's HSD test).

**FIGURE 7:**
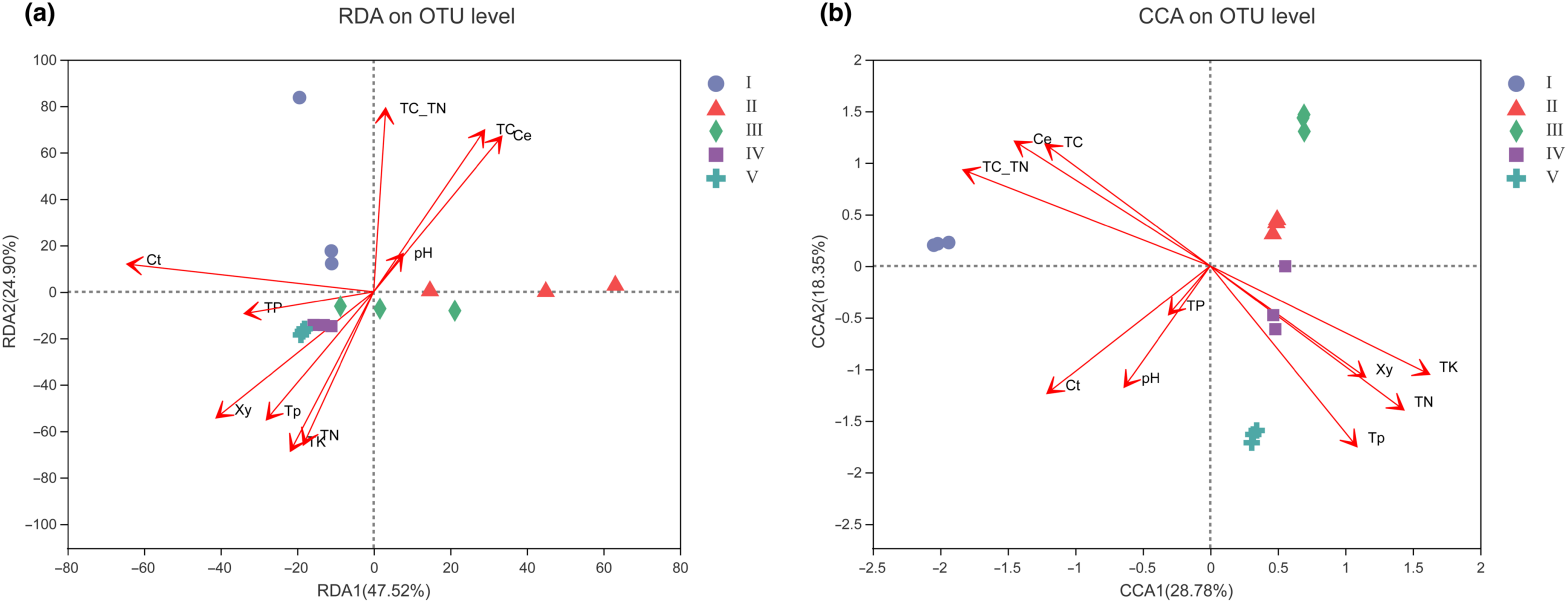
Canonical correlation analysis of relative abundances of bacterial communities (a) and redundancy analysis of relative abundances of fungal communities (b) assessed at OTU levels.

**FIGURE 8:**
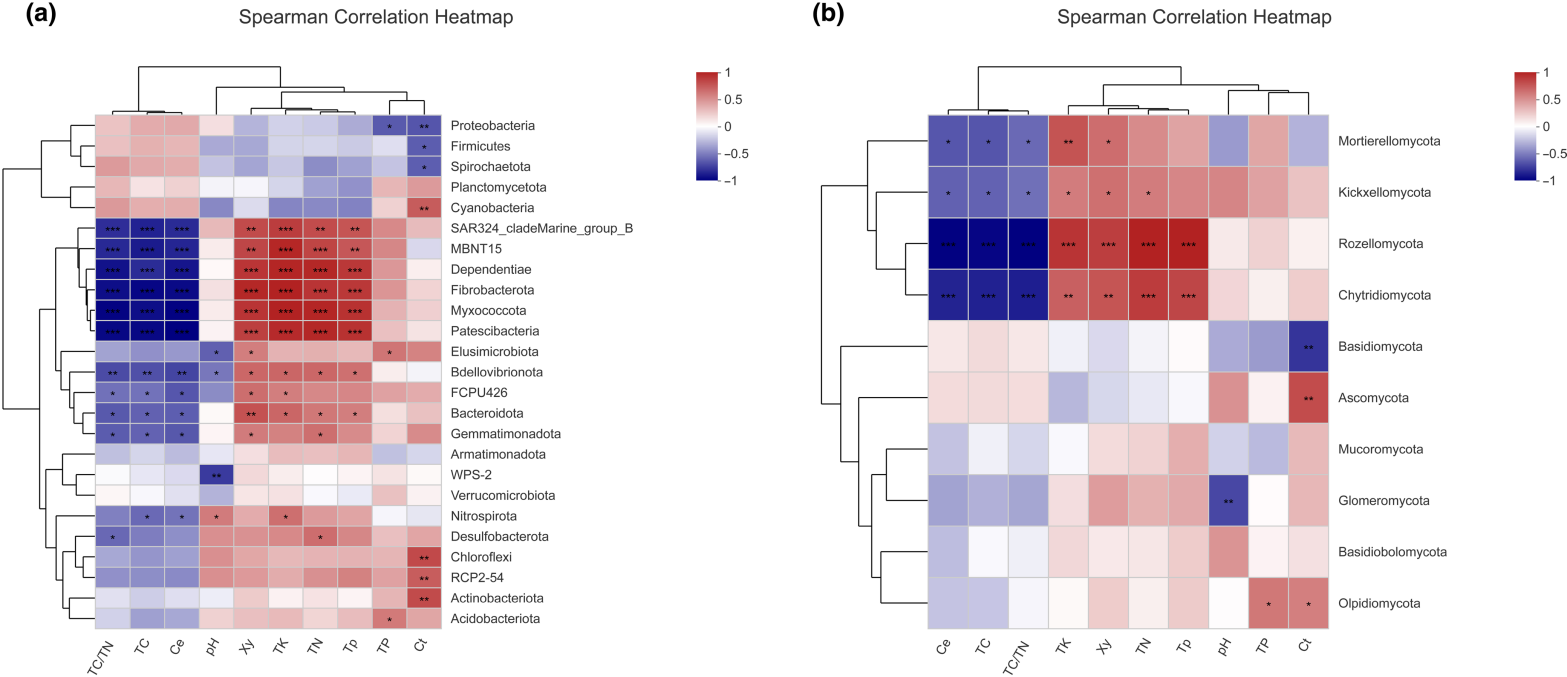
Correlation heatmap of the top 50 bacterial (a) and fungal phyla (b) with deadwood properties. *R* values are indicated on the right side of the legend with different colors. *p < .05, **p < .01, and ***p < .001.

To determine the key factors affecting the structural composition and functional changes of microbial communities, we conducted a correlation analysis between the physical and chemical indexes of deadwood and microbial communities. We first used the species‐sample data (sample OTU table with 97% similarity) for DCA analysis, and found that the value of the first axis of lengths of the gradient in the bacterial community was <3.5, so RDA analysis was better. It was found that the two axes of RDA analysis explained 72.42% of the changes in the bacterial community, among which TC, TN, TC/TN, TK, *C*_t_, *C*_e_, and *X*_y_ were significantly correlated with the changes in bacterial community structure (*p* < .01), and *T*_p_ was also significantly correlated with them (*p* < .05) (Table S2, Figure 7a). Spearman's correlation heatmap showed that the abundance of *SAR324_cladeMarine_group_B*, *MBNT15*, *Dependentiae*, *Fibrobacterota*, *Myxococcota*, and *Patescibacteria* was significantly negatively correlated with TC, *C*_e_, and TC/TN (*p* < .01), and was positively correlated with *X*_y_, TK, TN, and *T*_p_ (*p* < .01). The abundance of *Bacteroidota* was negatively correlated with TC, *C*_e_, and TC/TN (*p* < .05), positively correlated with TK, TN, and *T*_p_ (*p* < .05), and positively correlated with *X*_y_ (*p* < .01). In addition, the abundance of *Proteobacteria* was significantly negatively correlated with *C*_t_ (*p* < .01). The abundance of *Firmicutes* was significantly negatively correlated with *C*_t_ (*p* < .05), while the abundance of *Actinobacteriota* was significantly positively correlated with *C*_t_ (*p* < .01) (Figure 8a).

The value of the first axis of the lengths of the gradient in the fungal community was greater than 3.5, so CCA analysis was used. The results of CCA showed that TC, TN, TC/TN, TK, *C*_t_, *C*_e_, and *T*_p_ were significantly correlated with changes in fungal community structure (*p* < .01), and TC/TN and *T*_p_ (*r* ^2^ > .9) had a greater impact on the changes. *X*_y_ was significantly correlated with changes in fungal community structure (*p* < .05) (Table S3, Figure 7b). Spearman correlation heatmap showed that the abundance of *Rozellomycota* and *Chytridiomycota* was negatively correlated with TC, TC/TN and *C*_e_ at the phylum level (*p* < .01). However, it was positively correlated with TK, TN, *X*_y_, and *T*_p_ (*p* < .01). The abundance of *Mortierellomycota* and *Kickxellomycota* was negatively correlated with TC, TC/TN, and *C*_e_ (*p* < .05), but positively correlated with *X*_y_ (*p* < .05). The abundance of *Mortierellomycota* was positively correlated with TK (*p* < .01), and the abundance of *Kickxellomycota* was positively correlated with TN and TK (*p* < .05). *C*_t_ was positively correlated with *Ascomycota* abundance and negatively correlated with *Basidiomycota* abundance (*p* < .01) (Figure 8b).

## 4 DISCUSSION

### 4.1 Taxa‐specific changes in the microbial community of deadwood of *P. massoniana* at different stages of decomposition

Deadwood provides habitats for different species of microorganisms to occupy their respective ecological niches (Yuan et al., 2017). The decomposition of deadwood is influenced by numerous biological and non‐biological factors, while the diameter class and decay class of deadwood also influences microbial diversity (Mieszkin et al., 2021). In this study, the community diversity and richness of bacteria and fungi were the highest in decay class V deadwood and the lowest in decay class I. Moreover, the community composition of decay class I deadwood significantly different from that of other decay classes. This may be due to the density of wood, soluble wood extract, cellulose and lignin content, and other factors in the early stage of wood decomposition limit the propagation of dominant microbial communities related to wood decomposition (Prewitt et al., 2014). Studies have reported that the increase in fungal species richness in wood during the decay is associated with the gain of mycorrhizal and some soil‐saprotroph species (Mäkipää et al., 2017). The continuous work, propagation and complex intraspecific and interspecific interactions of the microorganisms colonized on the deadwood could be an important reason for the increase of microbial community diversity and richness in the process of decomposition. In this study, the species composition of bacterial communities changed less than that of fungal communities during decomposition, which was similar to the results of Kielak et al. (2016). In the fungal communities, *Ascomycota* and *Basidiomycota* are the main microbial groups that degrade organic carbon in deadwood which is difficult to be decomposed (Van der Wal et al., 2013). We found that *Ascomycota* dominated in I decay classes deadwood, and *Basidiomycota* dominated in II, III, and IV decay classes deadwood. The dominant fungal taxa gradually changed from *Ascomycota* to *Basidiomycota* and finally to *Ascomycota*, this reflects the coordination and succession of fungi during the decomposition of deadwood, which is similar to the results of Qi et al. (2023).

LEfSe analysis could provide new insights into the response of microbial communities to niche changes in the process of deadwood decomposition. We found that the abundance of *Actinobacteriota*, *Firmicutes*, and *Proteobacteria*, the dominant bacterial phyla, was significantly enriched in decay classes I, II, and III deadwood, respectively. The abundance of *Ascomycota*, the dominant fungal phyla, was significantly enriched in decay classes I and V deadwood, respectively, and the abundance of *Basidiomycota* and *Mortierellomycota* was significantly enriched in decay classes II and III deadwood. This suggested that the ecological niches of bacteria and fungi would also change as decomposition progresses, which was similar to the results of Jiang et al. (2021). We also found that the dominant phylum *Firmicutes* and *Proteobacteria* were significantly negatively correlated with the content of condensed tannins, while the content of condensed tannins was lower in decay classes II and III deadwood, so it would be that the content of condensed tannins affected the survival of *Firmicutes* and *Proteobacteria*. In addition, we found that some microbial taxa (*Myxococcota*, *Patescibacteria*, and *Chytridiomycota*) showed significantly enriched in class V deadwood, due to their low abundance (less than 1%), they likely contributed less to the deadwood decomposition (Hu et al., 2017). The reason for the significant enrichment, we speculate that it may be related to some nutrients in the deadwood. In summary, it could be seen that the changes in microbial community composition during the decomposition were closely related to the physicochemical properties of deadwood, and nutrients in deadwood could even dominate the survival of microbial species, which provided a reference for future studies on the role of microbial communities in the decomposition process of deadwood.

### 4.2 Changes in functional potential of the microbial communities in different decomposition stages of *P. massoniana* deadwood

In this study, the PICRUSt2 tool was used to predict and analyze the relative abundances of different bacterial metabolic pathways based on KEGG and eggNOG databases, and it was found that the relative abundances of functions related to amino acid transport and metabolism and carbohydrate metabolism were relatively important in bacterial communities. Studies have shown that bacteria participate in the decomposition of carbohydrates (such as lignin and cellulose) and N fixation in wood (Hervé et al., 2016; Hoppe et al., 2014), which is similar to our findings. Sarah et al. (2016) show that low nitrogen can limit the decomposition of fungi to deadwood. Our study found that the relative abundance of bacteria with functions related to carbohydrate metabolism in the early stage of deadwood decomposition was higher than that in the late stage, which might be due to the low N content of deadwood in the early decomposition stage, that limits the decomposing action by fungi. Therefore, the decomposition of carbohydrates in the early decomposition stage may be mainly completed by the bacterial community. We also found that saprotrophic fungi and saprophyte‐symbiotic fungi were dominant in the decomposition process. With the progress of decomposition, the relative abundance of saprotrophic fungi gradually decreased, and the relative abundance of Saprophyte‐Symbiotic fungi decreased from low to high and finally to decrease, which could be related to the occurrence of succession in the fungal community. However, since FunGuild only predicted the biological functions of fungi based on the known literature, and the same fungus had different biological functions on different hosts in different environments, whether the known saprophytic fungi were also saprophytic in deadwood needs further research. The functional predictions derived from amplicon sequencing data can initially assess the presence of notable variances in microbial community metabolic pathways among distinct sample groups, offering a basis for further research. However, due to the limitation of amplicon sequencing data in revealing the functional composition of the microbial community, it can solely anticipate functional alterations within the microbial community, with the predicted information being considerably restricted (Boshuizen & Te Beest, 2023). Consequently, for the subsequent research phase, we recommend employing more precise sequencing techniques, such as shotgun metagenome sequencing, to investigate shifts in microbial community function within deadwood.

### 4.3 Driving factors of microbial community changes in *P. massoniana* deadwood at different decomposition stages

Nutrient cycling in deadwood is important for the maintenance of the carbon cycle and habitat of decomposing organisms in forest ecosystems (Harmon et al., 1986). We found significant differences in physicochemical properties among different decomposition decay classes of *P. massoniana* deadwood except for total phosphorus (TP) content, which was similar to the results of Smyth et al. (2016). A large number of studies have shown that some nitrogen‐fixing bacteria, ectomycorrhiza or some fungi can affect the N content in deadwood (Bentzon‐Tilia et al., 2015; Rinne et al., 2016). In our study, the total nitrogen content of deadwood gradually increased from decay classes I–V, consistent with the results of Pastorelli et al. (2021), which might be due to the effect of nitrogen‐fixing bacteria and the transport of some ectomycorrhiza or some fungi. The total organic carbon content gradually decreased from decay classes I–V, consistent with the study of Yang et al. (2021), which may be attributed to the decomposer reducing the carbon content in the deadwood through respiration. Condensed tannins, phenolics, and pH also influence the microbial decomposition of deadwood during decomposition (Tláskal et al., 2017; Wang et al., 2021). Phenolic compounds can control enzyme activity and carbon and nitrogen utilization, and alter microbial community composition and respiration rates (Stanek et al., 2021). We found that the changes in bacterial community and fungal community structure were significantly correlated with the total phenolic content and condensed tannin content. It indicated that the total phenolic content and condensed tannin content of deadwood had significant effects on the composition of bacterial and fungal communities, which was consistent with the results of Stanek et al. (2021) It has been demonstrated that the pH of wood also affects the microbial community (Sarah et al., 2016), but we found that there was no significant correlation between them, which might be due to the different species of wood, the exact reason still needs further study.

In addition, the surrounding environmental factors (temperature, humidity, moisture, etc.) and epiphytes would also affect the nutrient content and microbial community in the deadwood (Přívětivý et al., 2016). Brischke and Alfredsen (2020) find that moisture is a key parameter and governing factor for fungal growth and wood decomposition. Zhan et al. (2020) show that low temperature and moist environments can disrupt the physical structure of litter and degrade the components that are difficult to decompose (cellulose and lignin), thus improving the efficiency of substrate utilization by microorganisms. Our study focused on the nutrient content and microbial community dynamics in deadwood during decomposition, while the influence of surrounding environmental factors on the decomposition of deadwood would be studied in the next step.

### 4.4 Study limitations

Amplicon sequencing technology can help us understand the diversity and species abundance changes of environmental microorganisms (Haider et al., 2024). However, with the development of sequencing technology, amplicon sequencing has some technical challenges. Biases in estimating microbial community abundance can arise from factors such as PCR primer selection, PCR template concentration, amplification conditions, pooling of multiple barcodes and sequencing (Raju et al., 2018). Furthermore, our sampling of *P. massoniana* deadwood samples using the “space‐for‐time” research method was conducted only once, neglecting the potential influence of environmental changes and time on deadwood decomposition. Therefore, we recommend that future research endeavors adopt more precise metagenomic sequencing technologies to sample deadwood at different times and increase the number of samples. This approach will enrich our understanding of the deadwood decomposition process.

## 5 CONCLUSIONS

The results suggest that the microbial community structure of the early decomposition (decay class I) was significantly different from the other decay classes, while the diversity and richness of the microbial community were the highest in the late decomposition (decay class V). *Proteobacteria* is the dominant bacteria phylum of all decay classes deadwood, *Ascomycota* is the dominant fungal phylum of decay classes I and V deadwood, and *Basidiomycota* is the dominant fungal phylum of decay classes II, III, and IV deadwood. At the phylum to genus level, most bacterial and fungal taxa are mainly enriched in decay classes I and V deadwood, while the enriched microbial taxa might be related to the nutrient composition of deadwood. In the early stages of decomposition, the relative abundance of the bacterial functional group involved in carbohydrate metabolism was greater than in the later stages. The relative abundance of saprophytic fungi gradually decreased with the progress of decomposition, and the comparative abundance of mixed saprophytic‐symbiotic fungi changed from low to high and then decreased. In addition, there are significant differences in physicochemical properties except for total phosphorus content at different decay classes of deadwood. Total carbon, total nitrogen, C/N ratio, total potassium, total phenol, condensed tannins, lignin, and cellulose are the key environmental variables that affect the changes in microbial community structure and function, among which C/N ratio has the greatest impact on the microbial community. In conclusion, our study contributes to a better understanding of the dynamics of microbial communities during deadwood decomposition.

## AUTHOR CONTRIBUTIONS

**Bingyang Shi:** Conceptualization (equal); data curation (lead); methodology (lead); resources (equal); writing – original draft (lead). **Xiurong Wang:** Conceptualization (equal); funding acquisition (lead); writing – review and editing (lead). **Shuoyuan Yang:** Investigation (equal); resources (equal). **Hongmei Chen:** Investigation (equal); resources (equal). **Yang Zhao:** Funding acquisition (equal); writing – review and editing (equal). **Junjie Shen:** Investigation (equal); resources (equal). **Meixuan Xie:** Investigation (equal); resources (equal). **Bufang Huang:** Investigation (equal); resources (equal).

## FUNDING INFORMATION

This research was funded by the National Natural Science Foundation of China, “Landscape Adaptation Evaluation of Bryophytes in Karst Areas and its Landscape Theory” (31960328), National Natural Science Foundation of China (32060353), and the Science and Technology Talent Platform Project of Guizhou Province ([2018] 5261).

## CONFLICT OF INTEREST STATEMENT

The authors declare that the research was conducted in the absence of any commercial or financial relationships that could be construed as a potential conflict of interest.

## Supporting information


Appendix S1.

Appendix S2.

## Data Availability

The data that support the findings of this study are available in the Supporting information of this article. The raw reads were uploaded to the NCBI Sequence Read Archive database (Accession Number: PRJNA905865).

## References

[ece311210-bib-0001] Baldrian, P. (2017). Microbial activity and the dynamics of ecosystem processes in forest soils. Current Opinion in Microbiology, 37, 128–134. 10.1016/j.mib.2017.06.008 28689057

[ece311210-bib-0002] Baldrian, P. , Zrůstov'a, P. , Tl'askal, V. , Davidov'a, A. , Merhautov'a, V. , & Vrska, T. (2016). Fungi associated with decomposing deadwood in a natural beech‐dominated forest. Fungal Ecology, 23, 109–122. 10.1016/j.funeco.2016.07.001

[ece311210-bib-0003] Bentzon‐Tilia, M. , Severin, I. , Hansen, L. H. , & Riemann, L. (2015). Genomics and ecophysiology of heterotrophic nitrogen‐fixing bacteria isolated from estuarine surface water. MBio, 6(4), e00929. 10.1128/mbio.00929-15 26152586 PMC4495170

[ece311210-bib-0004] Blois, J. L. , Williams, J. W. , Fitzpatrick, M. C. , Jackson, S. T. , & Ferrier, S. (2013). Space can substitute for time in predicting climate‐change effects on biodiversity. PNAS, 110, 9374–9379. 10.1073/pnas.1220228110 23690569 PMC3677423

[ece311210-bib-0005] Boshuizen, H. C. , & Te Beest, D. E. (2023). Pitfalls in the statistical analysis of microbiome amplicon sequencing data. Molecular Ecology Resources, 23(3), 539–548. 10.1111/1755-0998.13730 36330663

[ece311210-bib-0006] Brischke, C. , & Alfredsen, G. (2020). Wood‐water relationships and their role for wood susceptibility to fungal decay. Applied Microbiology and Biotechnology, 104(9), 3781–3795. 10.1007/s00253-020-10479-1 32144473 PMC8326242

[ece311210-bib-0007] Chang, C. H. , Wu, F. Z. , Yang, W. Q. , Tan, B. , Xiao, S. , Li, J. , & Gou, X. L. (2014). The dynamics of microbial community structure at different stages of log decay in an alpine forest of western Sichuan. Chinese Journal of Applied & Environmental Biology, 20(6), 978–985. 10.17521/cjpe.2015.0002

[ece311210-bib-0008] Chen, S. , Zhou, Y. , Chen, Y. , & Gu, J. J. B. (2018). fastp: An ultra‐fast all‐in‐one FASTQ preprocessor. Bioinformatics, 34, i884–i890. 10.1093/bioinformatics/bty560 30423086 PMC6129281

[ece311210-bib-0009] Cui, B. K. , Yuan, H. S. , Zhou, L. W. , He, S. H. , & Wei, Y. L. (2019). Species diversity of wood rot fungi on fallen conifers in the large and small Hinggan Mountains. Biodiversity Science, 27(8), 887–895. 10.17520/biods.2019053

[ece311210-bib-0010] Douglas, G. M. , Maffei, V. J. , Zaneveld, J. R. , Yurgel, S. N. , Brown, J. R. , Taylor, C. M. , Huttenhower, C. , & Langille, M. J. (2020). PICRUSt2 for prediction of metagenome functions. Nature Biotechnology, 38, 685–688. 10.1038/s41587-020-0548-6 PMC7365738 32483366

[ece311210-bib-0011] Edgar, R. C. (2013). UPARSE: Highly accurate OTU sequences from microbial amplicon reads. Nature Methods, 10, 996–998. 10.1038/nmeth.2604 23955772

[ece311210-bib-0012] Eichlerová, I. , Homolka, L. , Žifčáková, L. , Lisá, L. , Dobiášová, P. , & Baldrian, P. (2015). Enzymatic systems involved in decomposition reflects the ecology and taxonomy of saprotrophic fungi. Fungal Ecology, 13, 10–22. 10.1016/j.funeco.2014.08.002

[ece311210-bib-0013] Fan, H. , Sun, L. W. , Yang, L. G. , Zhou, J. C. , Yin, P. P. , Li, K. , Xue, Q. , Li, X. , & Liu, Y. J. (2018). Assessment of the bio active phenolic composition of *Acer truncatum* seed coat as a byproduct of seed oil. Industrial Crops and Products, 118, 11–19. 10.1016/j.indcrop.2018.03.030

[ece311210-bib-0014] Gu, Y. , Wang, J. , Cai, W. , Li, G. , Mei, Y. , & Yang, S. (2021). Different amounts of nitrogen fertilizer applications alter the bacterial diversity and community structure in the rhizosphere soil of sugarcane. Frontiers in Microbiology, 12, 721441. 10.3389/fmicb.2021.721441 34616383 PMC8489880

[ece311210-bib-0015] Guo, Q. Q. , Pan, J. W. , Li, H. E. , Gao, C. , Sun, X. G. , & Yang, J. (2019). Eco‐stoichiometric characteristics of soil carbon, nitrogen and phosphorus in *Pinus massoniana* plantation in Plateau mountainous areas, Guizhou Provience. Journal of Soil and Water Conservation, 4, 293–298.

[ece311210-bib-0016] Haider, D. , Hall, M. W. , LaRoche, J. , & Beiko, R. G. (2024). Mock microbial community meta‐analysis using different trimming of amplicon read lengths. Environmental Microbiology, 26(1), e16566. 10.1111/1462-2920.16566 38149467

[ece311210-bib-0017] Harmon, M. E. , Franklin, J. F. , Swanson, F. J. , Sollins, P. , Gregory, S. V. , Lattin, J. D. , Anderson, N. H. , Cline, S. P. , Aumen, N. G. , Sedell, J. R. , Lienkaempeer, G. W. , & Cromack, K. (1986). Ecology of coarse woody debris in temperate. Ecosystems, 15, 133–302.

[ece311210-bib-0018] Hervé, V. , Ketter, E. , Pierrat, J. C. , Gelhaye, E. , & Frey‐Klett, P. (2016). Impact of *Phanerochaete chrysosporium* on the functional diversity of bacterial communities associated with decaying wood. PLoS One, 11(1), e0147100. 10.1371/journal.pone.0147100 26824755 PMC4732817

[ece311210-bib-0019] Hoppe, B. , Kahl, T. , Karasch, P. , Wubet, T. , Bauhus, J. , Buscot, F. , & Krüger, D. (2014). Network analysis reveals ecological links between N‐fixing bacteria and wood‐decaying fungi. PLoS One, 9(2), e88141. 10.1371/journal.pone.0088141 24505405 PMC3914916

[ece311210-bib-0020] Hoppe, B. , Krüger, D. , Kahl, T. , Arnstadt, T. , Buscot, F. , Bauhus, J. , & Wubet, T. (2015). A pyrosequencing insight into sprawling bacterial diversity and community dynamics in decaying deadwood logs of Fagus sylvatica and Picea abies. Scientific Reports, 5(1), 9456. 10.1038/srep09456 25851097 PMC4389208

[ece311210-bib-0021] Hu, H. , Chen, X. J. , Hou, F. J. , & Cheng, Y. X. (2017). Bacterial and fungal community structures in loess plateau grasslands with different grazing intensities. Frontiers in Microbiology, 8, 606. 10.3389/fmicb.2017.00606 28439265 PMC5383705

[ece311210-bib-0022] Jiang, S. , Xing, Y. J. , Liu, G. C. , Hu, C. Y. , Wang, X. C. , Yan, G. Y. , & Wang, Q. G. (2021). Changes in soil bacterial and fungal community composition and functional groups during the succession of boreal forests. Soil Biology and Biochemistry, 161, 108393. 10.1016/J.SOILBIO.2021.108393

[ece311210-bib-0023] Kielak, A. M. , Scheublin, T. R. , Mendes, L. W. , & Kuramae, E. E. (2016). Bacterial community succession in pine‐wood decomposition. Frontiers in Microbiology, 7, 231. 10.3389/fmicb.2016.00231 26973611 PMC4771932

[ece311210-bib-0024] Li, M. , Ding, G. J. , Sun, X. G. , Luo, X. M. , & Zhang, R. B. (2016). Plant diversity and soil enzyme activity in 4 typical communities of *Pinus massoniana* in Guizhou. Journal of Forest and Environment, 36(4), 434–441. 10.13324/j.cnki.jfcf.2016.04.009

[ece311210-bib-0025] Liu, A. , Li, Y. , Wang, Q. , Zhang, X. , Xiong, J. , Li, Y. , & Sun, Y. (2022). Analysis of microbial diversity and community structure of rhizosphere soil of Cistanche salsa from different host plants. Frontiers in Microbiology, 13, 971228. 10.3389/fmicb.2022.971228 36046015 PMC9421434

[ece311210-bib-0026] Liu, C. S. , Zhao, D. F. , Ma, W. J. , Guo, Y. D. , Wang, A. J. , Wang, Q. L. , & Lee, D. J. (2016). Denitrifying sulfide removal process on high‐salinity wastewaters in the presence of *Halomonas* sp. Applied Microbiology and Biotechnology, 100(3), 1421–1426. 10.1007/s00253-015-7039-6 26454867

[ece311210-bib-0027] Liu, S. Q. , Yang, R. , Peng, X. D. , Hou, C. L. , Ma, J. B. , & Guo, J. R. (2022). Contributions of plant litter decomposition to soil nutrients in ecological tea gardens. Agriculture, 12, 957. 10.3390/agriculture12070957

[ece311210-bib-0028] Luo, X. , & Yu, C. (2021). Diversity of endophytic fungi from *Pinus massoniana* in Guizhou Province, southwestern China. Mycosystema, 40(3), 531–546. 10.13346/j.mycosystema.200251

[ece311210-bib-0029] Magnússon, R. Í. , Tietema, A. , Cornelissen, J. H. C. , & Kalbitz, K. (2016). Tamm review: sequestration of carbon from coarse woody debris in forest soils. Forest Ecology and Management, 377, 1–15. 10.1016/j.foreco.2016.06.033

[ece311210-bib-0030] Magoc, T. , & Salzberg, S. L. (2011). FLASH: Fast length adjustment of short reads to improve genome assemblies. Bioinformatics, 27, 2957–2963. 10.1093/bioinformatics/btr507 21903629 PMC3198573

[ece311210-bib-0031] Mäkipää, R. , Rajala, T. , Schigel, D. , & Ovaskainen, O. (2017). Interactions between soil‐ and dead wood‐inhabiting fungal communities during the decay of Norway spruce logs. The ISME Journal, 11, 1964–1974. 10.1038/ismej.2017.57 28430188 PMC5563949

[ece311210-bib-0032] Mieszkin, S. , Richet, P. , Bach, C. , Lambrot, C. , Augusto, L. , Buée, M. , & Uroz, S. (2021). Oak decaying wood harbors taxonomically and functionally different bacterial communities in sapwood and heartwood. Soil Biology and Biochemistry, 155, 8160. 10.1016/j.soilbio.2021.108160

[ece311210-bib-0033] Moll, J. , Kellner, H. , Leonhardt, S. , Stengel, E. , Dahl, A. , Bässler, C. , Buscot, F. , Hofrichter, M. , & Hoppe, B. (2018). Bacteria inhabiting deadwood of 13 tree species are heterogeneously distributed between sapwood and heartwood. Environmental Microbiology, 20, 3744–3756. 10.1111/1462-2920.14376 30109768

[ece311210-bib-0034] Ni, X. W. , Ning, C. , Yan, W. D. , Liu, Z. Z. , Chen, Y. , & Ning, X. B. (2017). Soil nutrients status of masson pine and slash pine plantations in Guizhou Longli forest farm. Journal of Central South University of Forestry & Technology, 09, 49–56.

[ece311210-bib-0035] Pastorelli, R. , Paletto, A. , Agnelli, A. E. , Lagomarsino, A. , & De Meo, I. (2021). Microbial diversity and ecosystem functioning in deadwood of black pine of a temperate forest. Forests, 12, 1418. 10.3390/f12101418

[ece311210-bib-0036] Peršoh, D. (2015). Plant‐associated fungal communities in the light of metaomics. Fungal Diversity, 75, 1–25. 10.1007/s13225-015-0334-9

[ece311210-bib-0037] Pioli, S. , Clagnan, E. , Chowdhury, A. A. , Bani, A. , Borruso, L. , Ventura, M. , & Brusetti, L. (2023). Structural and functional microbial diversity in deadwood respond to decomposition dynamics. Environmental Microbiology, 25(11), 2351–2367. 10.1111/1462-2920.16459 37403552

[ece311210-bib-0038] Prewitt, L. , Kang, Y. M. , Kakumanu, M. L. , & Williams, M. (2014). Fungal and bacterial community succession differs for three wood types during decay in a forest soil. Microbial Ecology, 68(2), 212–221. 10.1007/s00248-014-0396-3 24623527

[ece311210-bib-0039] Přívětivý, T. , Janík, D. , Unar, P. , Adam, D. , Král, K. , & Vrška, T. (2016). How do environmental conditions affect the deadwood decomposition of european beech (*Fagus sylvatica* L.)? Forest Ecology and Management, 381, 177–187. 10.1016/j.foreco.2016.09.033

[ece311210-bib-0040] Qi, L. L. , Yuan, J. , Zhang, W. J. , Liu, H. Y. , Li, Z. P. , Bol, R. , & Zhang, S. X. (2023). Metagenomics reveals the underestimated role of bacteria in the decomposition of downed logs in forest ecosystems. Soil Biology and Biochemistry, 187, 109185. 10.1016/j.soilbio.2023.109185

[ece311210-bib-0041] Raju, S. C. , Lagström, S. , Ellonen, P. , De Vos, W. M. , Eriksson, J. G. , Weiderpass, E. , & Rounge, T. B. (2018). Reproducibility and repeatability of six high‐throughput 16S rDNA sequencing protocols for microbiota profiling. Journal of Microbiological Methods, 147, 76–86. 10.1016/j.mimet.2018.03.003 29563060

[ece311210-bib-0042] Ren, Y. , Yu, G. , Shi, C. , Liu, L. , Guo, Q. , Han, C. , & Huang, H. (2022). Majorbio Cloud: A one‐stop, comprehensive bioinformatic platform for multiomics analyses. iMeta, 1(2), e12. 10.1002/imt2.12 PMC10989754 38868573

[ece311210-bib-0043] Ricker, M. C. , Lockaby, B. G. , Blosser, G. D. , & Conner, W. H. (2016). Rapid wood decay and nutrient mineralization in an old‐growth bottomland hardwood forest. Biogeochemistry, 127, 323–338. 10.1007/s10533-016-0183-y

[ece311210-bib-0044] Rinne, K. T. , Rajala, T. , Peltoniemi, K. , Chen, J. , Smolander, A. , Mäkipää, R. , & Treseder, K. (2016). Accumulation rates and sources of external nitrogen in decaying wood in a Norway spruce dominated forest. Functional Ecology, 31, 530–541. 10.1111/1365-2435.12734

[ece311210-bib-0045] Rinta‐Kanto, J. M. , Sinkko, H. , Rajala, T. , Al‐Soud, W. A. , Sørensen, S. J. , Tamminen, M. V. , & Timonen, S. (2016). Natural decay process affects the abundance and community structure of bacteria and Archaea in Picea abies logs. FEMS Microbiology Ecology, 92, 403–410. 10.1093/femsec/fiw087 27127195

[ece311210-bib-0046] Sarah, R. , Johnston, L. B. , & Andrew, J. W. (2016). Bacteria in decomposing wood and their interactions with wood‐decay fungi. FEMS Microbiology Ecology, 92, 11. 10.1093/femsec/fiw179 27559028

[ece311210-bib-0047] Shen, M. C. , Zhang, Y. Z. , Bo, G. D. , Yang, B. , Wang, P. , Ding, Z. Y. , & Yuan, X. L. (2022). Microbial responses to the reduction of chemical fertilizers in the rhizosphere soil of flue‐cured tobacco. Frontiers in Bioengineering and Biotechnology, 9, 812316. 10.3389/fbioe.2021.812316 35087808 PMC8787768

[ece311210-bib-0048] Shi, B. Y. , Wang, X. R. , Yang, S. Y. , & Chen, H. M. (2022). Addition *Pinus massonian*a fallen wood improved the growth of *Plagiomnium acutum* in a substrate cultivation. Scientific Reports, 12, 17755. 10.1038/s41598-022-21901-1 36272985 PMC9588075

[ece311210-bib-0049] Smyth, C. E. , Titus, B. , Trofymow, J. A. , Preston, C. M. , & Prescott, C. E. (2016). Patterns of carbon, nitrogen and phosphorus dynamics in decomposing wood blocks in Canadian forests. Plant and Soil, 409, 459–477. 10.1007/s11104-016-2972-4

[ece311210-bib-0050] Stanek, M. , Zubek, S. , & Stefanowicz, A. M. (2021). Differences in phenolics produced by invasive *Quercus rubra* and native plant communities induced changes in soil microbial properties and enzymatic activity. Forest Ecology and Management, 482, 118901. 10.1016/j.foreco.2020.118901

[ece311210-bib-0051] Tláskal, V. , Zrustová, P. , Vrška, T. , & Baldrian, P. (2017). Bacteria associated with decomposing dead wood in a natural temperate forest. FEMS Microbiology Ecology, 2, 157. 10.1093/femsec/fix157 29126113

[ece311210-bib-0052] Ulyshen, M. D. (2016). Wood decomposition as influenced by invertebrates. Biological Reviews, 91, 70–85. 10.1111/brv.12158 25424353

[ece311210-bib-0053] Van der Wal, A. , Geydan, T. D. , Kuyper, T. W. , & De Boer, W. (2013). A thready affair: linking fungal diversity and com m unity dynamics to terrestrial decom position processes. FEMS Microbiology Reviews, 37(4), 477–494. 10.1111/1574-6976.12001 22978352

[ece311210-bib-0054] Wang, Q. , Garrity, G. M. , Tiedje, J. M. , & Cole, J. R. (2007). Naïve Bayesian classifier for rapid assignment of rRNA sequences into the new bacterial taxonomy. Applied and Environmental Microbiology, 73, 5261–5267. 10.1128/AEM.00062-07 17586664 PMC1950982

[ece311210-bib-0055] Wang, S. , Wang, Y. X. , Liu, H. P. , Li, X. B. , Zhao, J. , Dong, Z. H. , Li, J. F. , Kaka, N. A. , Nazar, M. , & Shao, T. (2022). Using PICRUSt2 to explore the functional potential of bacterial community in alfalfa silage harvested at different growth stages. Chemical and Biological Technologies in Agriculture, 9, 98. 10.1186/s40538-022-00372-6

[ece311210-bib-0056] Wang, Z. , Yang, W. Q. , Tan, B. , Chang, C. H. , Wang, Q. , Jiang, Y. R. , & Cao, R. (2021). Effects of forest gap positions and epiphyties removal on total phenols and condensed tannins of fallen log in an alpine forest. Acta Ecologica Sinica, 41(4), 1451–1460. 10.5846/stxb202003230659

[ece311210-bib-0057] Yan, E. R. , Wang, X. H. , & Huang, J. J. (2005). Concept and classification of coarse woody debris in forest ecosystems. Acta Ecologica Sinica, 25(1), 158–167.

[ece311210-bib-0058] Yang, L. B. , Cui, F. X. , Huang, Q. Y. , Zhu, D. G. , & Xu, F. (2021). Analysis on bacterial diversity and composition of *Larixg melinii* fallen wood with different decomposition levels. Journal of Central South University of Forestry & Technology, 93, 100–182. 10.14067/j.cnki.1673-923x.2021.04.011

[ece311210-bib-0059] Yuan, J. , Zheng, X. , Cheng, F. , Zhu, X. , Hou, L. , Li, J. , & Zhang, S. (2017). Fungal community structure of fallen pine and oak wood at different stages of decomposition in the Qinling Mountains, China. Scientific Reports, 7(1), 13866. 10.1038/s41598-017-14425-6 29066754 PMC5654975

[ece311210-bib-0063] Zhan, P. , Liu, Y. , Wang, H. , Wang, C. , Xia, M. , Wang, N. , Cui, W. , Xiao, D. , & Wang, H. (2020). Plant litter decomposition in wetlands is closely associated with phyllospheric fungi as revealed by microbial community dynamics and co‐occurrence network. The Science of the total environment, 753, 142194. 10.1016/j.scitotenv.2020.142194 33207455

[ece311210-bib-0061] Zhou, F. , Wu, X. , Gao, Y. , Fan, S. , Zhou, H. , & Zhang, X. (2022). Diversity shifts in the root microbiome of cucumber under different plant cultivation substrates. Frontiers in Microbiology, 13, 878409. 10.3389/fmicb.2022.878409 35663868 PMC9159939

[ece311210-bib-0062] Zhu, Y. , Wang, Y. , Jiang, H. , Liu, W. , Zhang, S. , Hou, X. , & Chen, X. (2023). Transcriptome analysis reveals that PbMYB61 and PbMYB308 are involved in the regulation of lignin biosynthesis in pear fruit stone cells. The Plant Journal, 116(1), 217–233. 10.1111/tpj.16372 37382050

