# Supplementary material for: Changes and driving factors of microbial community composition and functional groups during the decomposition of Pinus massoniana deadwood
Source: Ecol Evol. 2024 Apr 1;14(4):e11210. doi: 10.1002/ece3.11210 (PMC10985386; doi:10.1002/ece3.11210)
Supplement: Supplementary file 1 — Appendix S1. [file ECE3-14-e11210-s002.docx]

**Changes and driving factors of microbial community composition and functional groups during the decomposition of Pinus massoniana deadwood**

**Bingyang Shi, Xiurong Wang^*^, Shuoyuan Yang, Hongmei Chen, Yang Zhao, Junjie Shen, Meixuan Xie, Bufang Huang**

Forestry College, Guizhou University, Guiyang550025, Guizhou China

*** Correspondence:** Xiurong Wang [xrwang@gzu.edu.cn](mailto:xrwang@gzu.edu.cn)

**F-Address:** College of Forestry, Guizhou University, Huaxi District, Guiyang, Guizhou, China

Supplementary Material

# Supplementary Figures and Tables

## Supplementary Tables

**Table. S1** Criteria for the decay classes of deadwood

| Decision criteria | Decay classes | | | | |
| --- | --- | --- | --- | --- | --- |
|  | Ⅰ | Ⅱ | Ⅲ | Ⅳ | Ⅴ |
| Needle | Present | Absent | Absent | Absent | Absent |
| Bark | No difference from living | Partial shedding | Partly present only on large branches | Absent | Absent |
| Branches and twig | Twigs present | Branches present | Large branches present | Only large branches stub present | Absent |
| Bole shape | Round | Round | Round | Round to oval | Round to flat |
| Structural integrity | Intact | Sapwood decayed, heartwood intact | The sapwood disappears and the heartwood is intact | Heartwood is rotten | Soften |
| Wood consistency | Solid | Solid | Semi-solid | Partially softened | Crushing to powder |
| Color of wood | Primary Colors | Primary Colors | Original color to fade | Original color to fade | Severe fading |
| Indirect measure | Fresh xylem, blade can be pierced 1-2 mm | Start of decomposition, blade penetrates 3 mm to 1 cm | Blade penetrates approx 2 cm | Severe decay, blade can penetrate 2-5cm | To pierce the woody body at will |

**Table S2** Results of RDA of bacterial communities assessed at OTU levels. The P values were based on 999 permutations.

| Environmental Variables | RDA1 | RDA2 | r^2^ | p_values |
| --- | --- | --- | --- | --- |
| TC | 0.4157 | 0.9095 | 0.7063 | 0.001 |
| TN | -0.3058 | -0.9521 | 0.5701 | 0.001 |
| TC/TN | 0.0801 | 0.9968 | 0.7528 | 0.001 |
| TP | -0.9557 | -0.2945 | 0.1495 | 0.399 |
| TK | -0.3382 | -0.9411 | 0.6354 | 0.002 |
| pH | 0.452 | 0.892 | 0.0342 | 0.878 |
| Tp | -0.4809 | -0.8768 | 0.4717 | 0.017 |
| Ct | -0.9899 | 0.1415 | 0.5256 | 0.006 |
| Ce | 0.4752 | 0.8799 | 0.6994 | 0.001 |
| Xy | -0.62 | -0.7846 | 0.5843 | 0.003 |

**Table S3** Results of CCA of fungal communities assessed at OTU levels. The P values were based on 999 permutations.

| Environmental Variables | CCA1 | CCA2 | r^2^ | p_values |
| --- | --- | --- | --- | --- |
| TC | -0.719 | 0.695 | 0.6148 | 0.004 |
| TN | 0.7173 | -0.6968 | 0.857 | 0.001 |
| TC/TN | -0.8899 | 0.4561 | 0.9059 | 0.001 |
| TP | -0.5446 | -0.8387 | 0.0593 | 0.667 |
| TK | 0.8391 | -0.5439 | 0.8005 | 0.001 |
| pH | -0.4743 | -0.8803 | 0.368 | 0.069 |
| Tp | 0.5299 | -0.8481 | 0.9056 | 0.001 |
| Ct | -0.6997 | -0.7144 | 0.6274 | 0.003 |
| Ce | -0.7673 | 0.6413 | 0.7629 | 0.003 |
| Xy | 0.7304 | -0.6831 | 0.5267 | 0.014 |

## Supplementary Figures


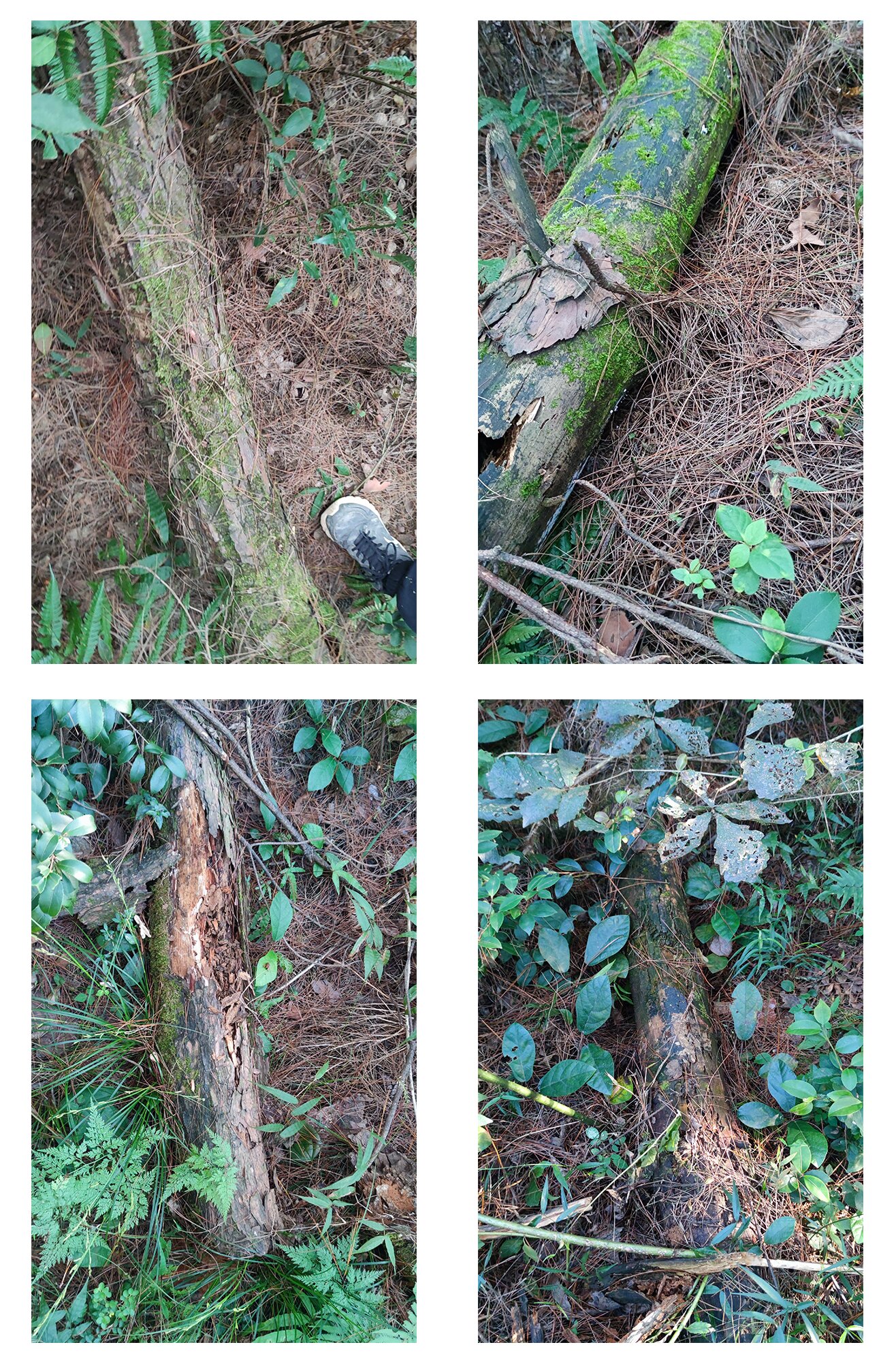


Figure S1 Site condition of deadwood sampling.


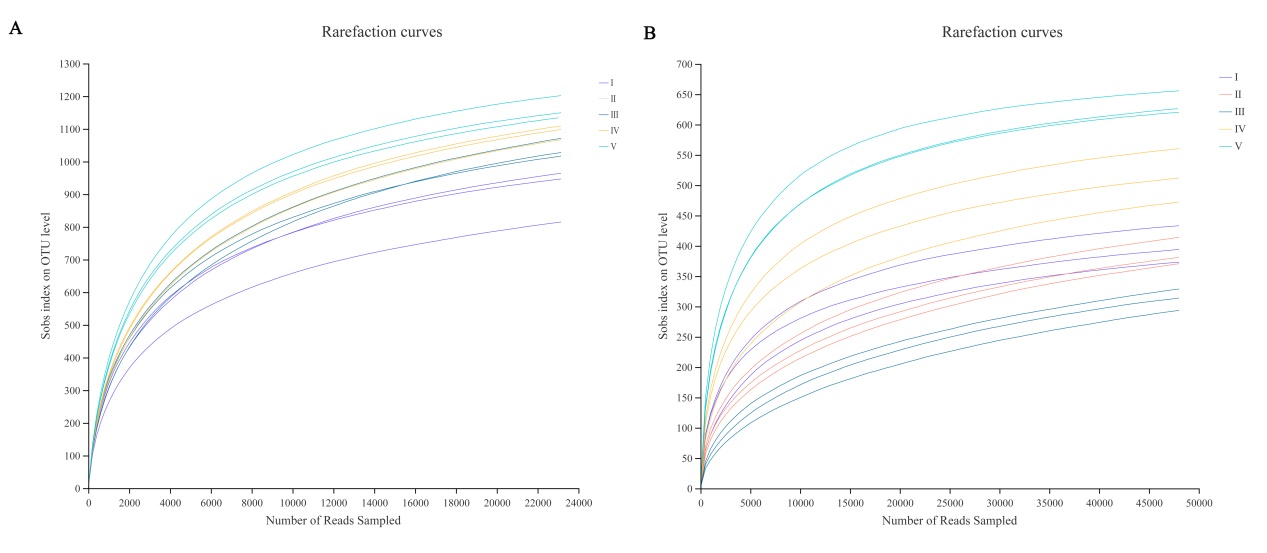


Figure S2 Rarefaction curves of bacterial (A) and fungal (B) diversity in dead wood with different decay classes.

Figure S3 Cladogram shows significant differences between bacterial (A) and fungal (B) enrichment groups. Taxa with significant differences in abundance between different successions are represented by colored dots. The inner to outer circle corresponds to the level of the phylum to the genus. The diameter of each circle is proportional to the abundance of the group. LDA score >3.5 for bacteria and fungi.
