# Supplementary material for: Changes and driving factors of microbial community composition and functional groups during the decomposition of Pinus massoniana deadwood
Source: Ecol Evol. 2024 Apr 1;14(4):e11210. doi: 10.1002/ece3.11210 (PMC10985386; doi:10.1002/ece3.11210)
Supplement: Supplementary file 2 — Appendix S2. [file ECE3-14-e11210-s001.docx]

| Deadwood | n | TC（g/kg） | TN（g/kg） | TC/TN | TP（g/kg） | TK（g/kg） | pH | Tp（g/kg） | Ct（g/kg） | Ce（g/kg） | Xy（g/kg） |
| --- | --- | --- | --- | --- | --- | --- | --- | --- | --- | --- | --- |
| Ⅰ | 1 | 48.43949 | 0.231099 | 209.6046 | 1.628791 | 0.277341 | 4.85 | 15.4713193 | 1.304631442 | 45.299246 | 431.4616 |
|  | 2 | 45.85443 | 0.277778 | 165.0759 | 1.613726 | 0.2778 | 4.865 | 14.0426076 | 1.350118591 | 43.189447 | 402.8575 |
|  | 3 | 43.55769 | 0.231252 | 188.3559 | 1.805659 | 0.296699 | 4.86 | 15.6265978 | 1.713327501 | 42.7031786 | 430.2465 |
| Ⅱ | 1 | 42.98077 | 0.416391 | 103.2221 | 1.17868 | 0.565331 | 4.835 | 17.9044257 | 0.413520316 | 39.3466157 | 446.1768 |
|  | 2 | 43.51974 | 0.370003 | 117.6199 | 1.613739 | 0.574731 | 4.95 | 18.6616902 | 0.452554216 | 32.8101462 | 416.5394 |
|  | 3 | 46.76471 | 0.369637 | 126.5152 | 1.135234 | 0.622052 | 4.95 | 17.4216028 | 0.523938573 | 27.1575643 | 413.7784 |
| Ⅲ | 1 | 41.86709 | 0.463423 | 90.3432 | 1.89166 | 0.864064 | 4.545 | 19.029405 | 0.616075959 | 18.4785179 | 506.5146 |
|  | 2 | 41.47059 | 0.415979 | 99.69398 | 1.764174 | 0.84262 | 4.565 | 17.3202782 | 0.710848644 | 26.6714 | 554.328 |
|  | 3 | 39.77419 | 0.369637 | 107.6034 | 1.45908 | 0.832764 | 4.565 | 17.3729779 | 0.43318172 | 17.3595687 | 458.974 |
| Ⅳ | 1 | 40.88235 | 0.554822 | 73.68557 | 1.408452 | 0.833314 | 4.535 | 22.9555601 | 1.302083333 | 15.892711 | 555.5648 |
|  | 2 | 38.77358 | 0.462963 | 83.75094 | 1.74449 | 0.872804 | 4.47 | 20.4873635 | 1.090393632 | 12.619612 | 559.0844 |
|  | 3 | 39.70588 | 0.463576 | 85.65126 | 1.761349 | 0.816302 | 4.495 | 21.2299991 | 1.529553152 | 9.82580891 | 553.1339 |
| Ⅴ | 1 | 34.81132 | 0.693985 | 50.16146 | 1.736883 | 1.188713 | 5.015 | 25.9485862 | 0.997569558 | 2.78270584 | 570.7299 |
|  | 2 | 37.87975 | 0.601057 | 63.02191 | 1.663186 | 1.159175 | 5.06 | 27.820219 | 1.16194626 | 4.30570265 | 528.2866 |
|  | 3 | 36.97452 | 0.555188 | 66.59816 | 1.95468 | 1.083218 | 5.025 | 26.9445402 | 1.602389016 | 2.66437945 | 587.541 |

**Raw data of environmental factors**
